# A Stated Preference Study to Explore Market-Based Instruments to Reduce Car Usage

**DOI:** 10.1007/s10640-025-01005-w

**Published:** 2025-06-05

**Authors:** Christopher Tate, Alberto Longo, Marco Boeri, Tim Taylor, Leandro Garcia, Ruth Hunter

**Affiliations:** 1 Centre for Public Health, School of Medicine, Dentistry and Biomedical Sciences, Institute of Clinical Science B, Royal Victoria Hospital, Grosvenor Road, Belfast, BT12 6BA UK; 2 Patient-Centred Outcomes, OPEN Health, 20 Old Bailey, London, EC4M 7AN UK; 3 School of Medicine, Dentistry and Biomedical Sciences, Queen’s University Belfast, Royal Victoria Hospital, Grosvenor Road, Belfast, BT12 6BA UK; 4 European Centre for Environment and Human Health, Department of Public Health and Sport Sciences, University of Exeter Medical School, Peter Lanyon, Penryn Campus, Penryn, TR10 9FE UK

**Keywords:** Car usage, Stated preference methods, Discrete choice experiment, Contingent valuation, Market-based instruments

## Abstract

Car dependency is becoming an increasingly difficult problem for policymakers to contend with, and requires targeted policy solutions that balance the need for greater urban mobility with reduced congestion. We investigated public preferences for welfare measures designed to encourage car use reduction and promote more sustainable urban environments. Cross-sectional survey data were obtained from *n* = 773 car owners living in Belfast, United Kingdom. A discrete choice experiment was used to elicit the willingness-to-pay (WTP) for a congestion charge that would finance policies to reduce car usage. A contingent valuation question assessed the willingness-to-accept (WTA) a monetary incentive to reduce car usage. WTP values were computed using a mixed logit model, and an interval data model was used to assess the factors that were correlated with WTA. We also calculated the benefit to the economy of reduced car usage. WTP for different policy measures ranged from £2.12 to £11. The highest WTP value was observed for improvements to public transport frequency, coverage, and connectivity. The median WTA value to reduce car usage by one day per week was £3. As a result of reduced emissions and road casualties, it was estimated that this intervention would generate benefits worth £3.83 m, however this was greatly outweighed by the costs involved.

**Supplementary Information:**

The online version contains supplementary material available at 10.1007/s10640-025-01005-w.

## Introduction

The proliferation of private vehicle use in urbanised economies, and coinciding growth in vehicle ownership [1], whilst facilitating the movement of goods and people, has engendered myriad negative externalities in the form of anthropogenic climate change and air pollution [2–4], congestion, traffic-related injuries and deaths [2, 5], noise pollution [6, 7], urban heat island effects [8], and car-oriented land use [9]. Growing private vehicle ownership (a corollary of car dependency) has created cities that are increasingly dependent on the car for transport, and reinforced a pattern of “compulsory consumption” that has exacerbated social inequalities [10].

Market-based instruments, such as congestion charges, have been examined as a means to tackle burgeoning car ownership and traffic congestion. However, relatively few studies have operationalised car owners’ willingness-to-pay (WTP) a congestion charge in terms of the impact it will have on quality of life in urban settings. As such, the present study considers car owners’ WTP a congestion charge as a function of hypothetical policy instruments that include reduced speed limits, improved public transport and cycling infrastructure, the creation of car-free green areas, and reduced public transport costs. In addition, we asked car owners to report the lowest amount they would be willing to accept as a monetary incentive to reduce their car usage by one day per week and used this to calculate the net benefit to the economy attributable to reduced emissions and traffic-related casualties.

The purpose of this analysis was to address the following research questions:

1. Are car owners willing to pay a congestion charge that will fund policy measures designed to reduce car dependency and improve the quality of life of residents living in an urban setting?
2. Does WTP a congestion charge increase proportionately with a change in the complexity and scope of hypothesized improvements to transport infrastructure in Belfast?
3. What is the net benefit to the economy attributable to reduced emissions and traffic-related casualties if car owners reduce their car usage by one day per week?

This paper is structured as follows: Sect. 2 discusses the current policy landscape with regard to car usage and provides a brief overview of the relevant literature; Sect. 3 outlines the data collection procedure and methodology employed; Sect. 4 presents the results of our analysis; and Sect. 5 discusses the findings of the present study within the context of existing research in this area and provides concluding remarks.

## Background and Policy Context

### Public Policy Strategies to Address Car Usage

A constellation of interconnected economic, political, and cultural forces have come to define modern transport infrastructure and in many cities this has resulted in the entrenchment of car-dependent mobility [11]. Further complications have arisen from the coupling of autonomy and mobility [12]. For instance, despite the financial burden car ownership places on families, particularly low-income households [13], policies that restrict car usage may be perceived as infringing on car owners’ liberties around freedom of movement [12]. Similarly, punitive measures that financially penalise car ownership risk indirectly worsening inequalities if they disproportionately affect those from disadvantaged backgrounds [14]. Thus, reducing car usage requires policies that balance equity considerations with the need to reduce the number of cars on the road and support the transition towards environmentally sustainable urban mobility. In search of a solution, policymakers have experimented with a variety of command-and-control policies as well as market-based instruments.

Command-and-control policies aim to restrict the use of vehicles through rules and standards enforceable by statutory bodies or government agencies. This may include emissions and technology standards on the supply side, or usage restrictions that directly impact car owners. Other examples include city centre access control strategies [15], banning the use of cars in certain areas and car-free days in cities [16–21], or alternating number plate systems [22]. Market-based instruments create economic disincentives tailored to “push” urban residents away from car use, and can include congestion charges or taxes which suppress demand by making it more expensive to drive or own a vehicle [23]. Revenue generated through road user taxation measures can then be used to improve the provision and quality of public transport services within cities [24–30], further increasing the opportunity cost of driving a car. Congestion charges have been proven to be effective at reducing congestion in cities such as London [31], Stockholm [32], and Milan [33, 34]. Restrictive policies can also have the added benefit of increasing the uptake of public transport, at least in the short-term, as evidenced by a study in China [35].

More recently, “pull” mechanisms such as public transport subsidies have been used to promote more sustainable modes of transport. Studies have shown that better public transport infrastructure contributes to lower levels of car dependency [36] and improved air quality [37]. In Northern Ireland, free and concessionary bus and rail travel is offered to anyone over the age of 60 years [38]. In England, people are eligible for a National Bus Pass when they reach the female retirement age (irrespective of sex), allowing them to avail of free public transport at off-peak times during weekdays, and all day during weekends and public holidays [39]. In Germany, discounted monthly rail tickets helped to increase rail travel following the COVID-19 pandemic [40]. Free public transport schemes have been trialled in other countries with some success [41–43]. Experimental studies have also been conducted to assess the impact of offering free public transport to car owners, and shown that offering free bus tickets can induce a modal shift away from cars [44, 45].

### Stated Preference Methods

Given that the aforementioned policies incur implementation costs that are typically covered by taxes, it is important to know whether societies are willing to spend the necessary amount of money to support them. For example, to assess the welfare improvements that a policy can achieve, researchers can rely on stated preference methods, such as discrete choice experiments (DCE) or contingent valuation (CV). These survey-based techniques, used to capture societal preferences for a given policy, have been widely used to derive non-market values for environmental policies based on respondents’ WTP for, or willingness-to-accept (WTA), a change in the provision of a non-market good.

WTP is grounded in the notion that individuals are willing to incur costs to enjoy environmental benefits such as reduced traffic congestion or improved air quality. Policymakers can estimate the value of these benefits to the public by, for example, eliciting WTP for a congestion charge that will fund improvements to public transport. However, a congestion charge will only reduce the number of cars on the road if the improvements to public transport compensate (in monetary terms) the value of the congestion charge. That is, car users will continue to use their cars if the utility gained through the improved transportation policies does not match or exceed their WTP. Previous studies have also examined WTP for improved air quality in cities across Europe [46, 47], Asia [48–55], North America [56], and Africa [57].

WTA measures how much individuals demand in compensation to give up their property rights or forgo certain activities. In the context of reducing car usage in urban areas, this approach can be applied to understand what incentives car owners are willing to accept for restricting their driving rights. As driving is viewed by many motorists as a right that they own, how receptive they are to a reduction to this right can be measured through the WTA approach.

Environmental evaluations employing stated preference methodologies predominantly focus on WTP. Applications of WTA have mostly been relegated to valuing decreases in respondents’ property rights, such as farmers being asked to restrict harmful practices in exchange for agri-environment subsidies [58, 59]; and individuals being asked to modify their health behaviours in exchange for compensation [60–62]. This is probably due to the National Oceanic and Atmospheric Administration panel’s recommendation favouring the use of WTP, viewing it as the “conservative choice” [63]. However, Whittington et al. [64] recently indicated that WTA is an appropriate measure for behaviour change, remarking that “if an individual has property or ownership rights for a particular good or service, losses of those rights would be best measured by a WTA question” (p.318). Only using one of these evaluation tools in the context of reducing car usage in urban areas is not going to present policymakers with sufficient information with which to make better decisions. Applying both WTP and WTA offers a more comprehensive approach, offering insights into the values attached to the benefits of reduced car usage, as well as the costs associated with relinquishing driving privileges. The complementarity of WTP and WTA means that harnessing both approaches can provide a better understanding of the value attached to reduced car usage.

### Policy Context

#### Car Usage Patterns

Belfast, the capital of Northern Ireland, is one of the most congested cities in the United Kingdom (UK) [65] which is partly attributable to high levels of car ownership. It has been estimated that the number of licensed vehicles per 1,000 people in Northern Ireland is 12% higher than the UK as a whole [66, 67]. In 2021, 69% of all journeys in Northern Ireland were made by car or van [68], whereas 59% of journeys were made by car or van in England [69]. By contrast, only 24% of journeys were made by walking, 2% by public transport (including bus and rail), and 1% by bicycle.

Public transport in Northern Ireland is not as heavily subsidised as in the rest of the UK [70], potentially contributing to underutilisation and therefore increased dependency on the car for travel. This is reflected in a recent report wherein a large proportion of Northern Ireland drivers indicated that poor public transport provision was an important factor that discouraged its use [71]. Travel data from Northern Ireland that were published in March 2023 [68] indicated that, on average, *n* = 583 journeys were made by car per person in 2021 (or *n* = 11.21 journeys per week). Not adjusting for seasonality, this equates to *n* = 1.60 journeys per person per day. The average journey length by car was 11.39 km. Thus, it was estimated that each person travelled 6646.59 km on average by car in Northern Ireland in 2021.

#### Existing Policies to Reduce Car Dependency in Belfast

The first *Regional Transportation Strategy* for Northern Ireland published in 2002 [72] acknowledged that the historically poor performance of public transport was compounded by a sustained reduction in walking and cycling, in addition to an increase in car use. Signalling its commitment to support a modal shift away from private cars, the Northern Irish government has invested heavily in new bus fleets, service routes, and rail infrastructure. For instance, £100 m was invested in a new rapid transit bus service (Glider) that has already yielded positive results with passenger journeys more than doubling between the period 2018/19 and 2019/20 [73]. In addition to this, there are various initiatives in place to support the use of bicycles [74], improvements to walking infrastructure [75], and investment in zero-emissions rapid transit bus services [76]. By the year 2030, the government aims to provide free public transport to children and young people [77]. Other plans focused on re-purposing land within the existing urban footprint to curb urban sprawl and reduce the burden of extended commuting are also in development [78]. Despite these ambitions, a meaningful reduction in car dependency is yet to materialise, and the problems that stem from this dependency are most acute in the capital city of Belfast where high levels of car ownership and congestion persist.

#### Characteristics of the Study Area

**Fig. 1:**
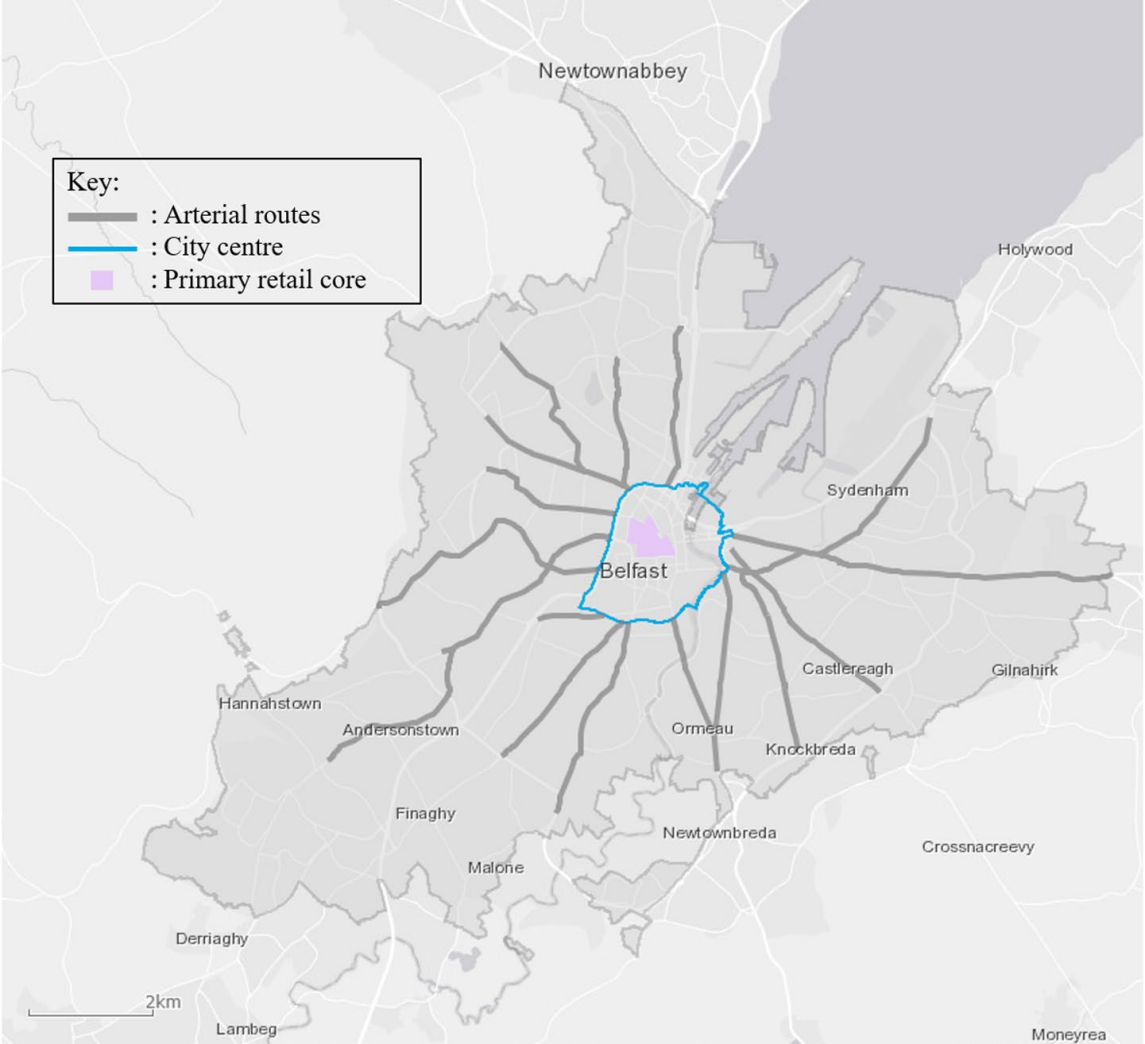
Map of the Belfast metropolitan area. *Source*: https://belfastcc.maps.arcgis.com/

Figure 1 shows the study area with a number of features highlighted, including the main arterial routes, city centre, and primary retail core. The Belfast metropolitan area covers approximately 960 km^2^, with a population density that ranges from 2,617 people per km^2^ in the most densely populated areas to 256 people per km^2^ in the least densely populated areas [79], and is characterised by a central urban core encircled by suburban sprawl and connected by a radial network of roads. The main arterial routes radiate outwards from the city centre in a design typical of the hub and spoke structure seen in most metropolitan regions in the UK to facilitate commuter flows into the centre. Commercial and retail activities are generally concentrated within the city centre, with a number of industrial and manufacturing sites located in the coastal areas to the North-East.

## Methodology

As previously alluded to, DCEs can be used to capture individuals’ preferences for different hypothetical policy instruments with varying characteristics [80]. Since no congestion charge currently exists in Belfast, we used the stated preference approach with an error component to capture correlation within individual observations. The multidimensional nature of the DCE enables researcher to compute distinct valuations from multiple choice sets with different attributes and levels that represent a hypothetical change in the provision of a non-market or environmental resource [81]. By comparison, CV questions designed to elicit WTA are appropriate in instances where an individual’s rights are diminished by a hypothetical policy or programme [64]. Hence, CV is a useful tool to gauge the level of compensation that is required to incentivise an individual to forfeit certain privileges, such as the right to drive.

### Survey Design

Cross-sectional online survey data were obtained from a sample of *n* = 773 car owners living in the Belfast metropolitan area aged ≥ 18 years. The online survey was developed using Qualtrics and distributed by Belfast-based polling and market research company LucidTalk between 18th and 25th February 2022. Additional information pertaining to the sampling method is included in Part A of the Appendix.

The survey was structured in 4 parts; part one asked participants to report their weekly car usage, rate public transport and walking infrastructure, and score the accessibility of local green spaces; part 2 contained 12 DCE choice tasks to elicit WTP for a congestion charge; part 3 was a CV question to capture WTA a monetary incentive to reduce car usage by one day per week; and part 4 covered respondents’ sociodemographic information. Sociodemographic data that were collected at the end of the survey included sex, age group, occupation, annual household income, highest qualification, years of education, nationality, and household composition. The survey instruments are described in Part A of the Appendix. The average response time for a completed survey was 17.8 min.

#### Discrete Choice Experiment

##### Attribute Selection

In light of earlier research that has documented the importance of qualitative methods to inform attribute development as a stepwise design process [82, 83], the DCE created for the present study was co-created and conceptualised (step one) with input from stakeholders as well as the general public [84], and went through a pre-testing phase (step two) that enabled us to gather feedback, simplify the text, refine some of the language around the descriptions of attributes, and remove redundant elements of the survey. We opted to use qualitative descriptions of attributes (excluding cost), drawing on examples from earlier studies that used descriptive labels [85–88]. This was done primarily due to the lack of clearly defined quantitative measures that could be identified during the early conceptualisation stages [85], and to reduce the cognitive burden on respondents.

The attributes and their respective levels were developed in two stages. The first stage was based on consolidating the findings from an umbrella review conducted by Cleland et al. [89] and a policy report produced by Queen’s University Belfast that presented a high-level overview of the policy landscape in Northern Ireland, synthesising evidence from various programmes and initiatives aimed at addressing car dependency [90]. The second stage was based on refining the set of attributes, and involved conducting focus groups, stakeholder workshops, and interviews with representatives from national and local public sector organisations, along with experts in the following areas: public health, urban planning, urban regeneration, architecture, engineering, active travel, public transport, inclusive mobility and transport, and environmental sustainability and development [84]. This process involved the use of system dynamics modelling and group model-building to develop a causal loop diagram [91]. The purpose of the group model-building workshops was to invite stakeholders to identify and examine priorities and strategies to reduce car dependency, as well as discuss the necessary steps for implementing these actions. Additionally, during one-to-one semi-structured interviews that lasted 45–60 min, participants were prompted to expound upon the reasons for, and consequences of, high levels of car usage in Belfast. Participants were also asked to propose a number of measures for reducing car dependency, and consider the potential impact of these initiatives, as well as the short-, medium-, and long-term opportunities for doing so.

In order to ensure that the proposed congestion charge levels were realistic and credible, we reviewed congestion charge values in other UK cities. We chose a price vector to capture the distribution of WTP considering car owners’ expected WTP, with insights from our stakeholders and experts during the consultation process mentioned above. Hence, the price vector includes values that are both higher and lower than, for example, the congestion charge in London (£15). For comparison, in other cities such as Birmingham (£8), Bristol (£9), and Oxford (up to £10) congestion charges are applied to vehicles that are non-compliant with clean air zone emissions standards.

After the survey was completed and the data were collected, the policy instruments explored in the DCE were presented to a Citizens’ Jury co-ordinated by Queen’s University Belfast, the purpose of which was to sense-check the proposed policy instruments [92]. Attendees at the event were in agreement that future policies should prioritise improving public transport and creating financial incentives to reduce car usage.

##### Choice Set Configuration

**Fig. 2:**
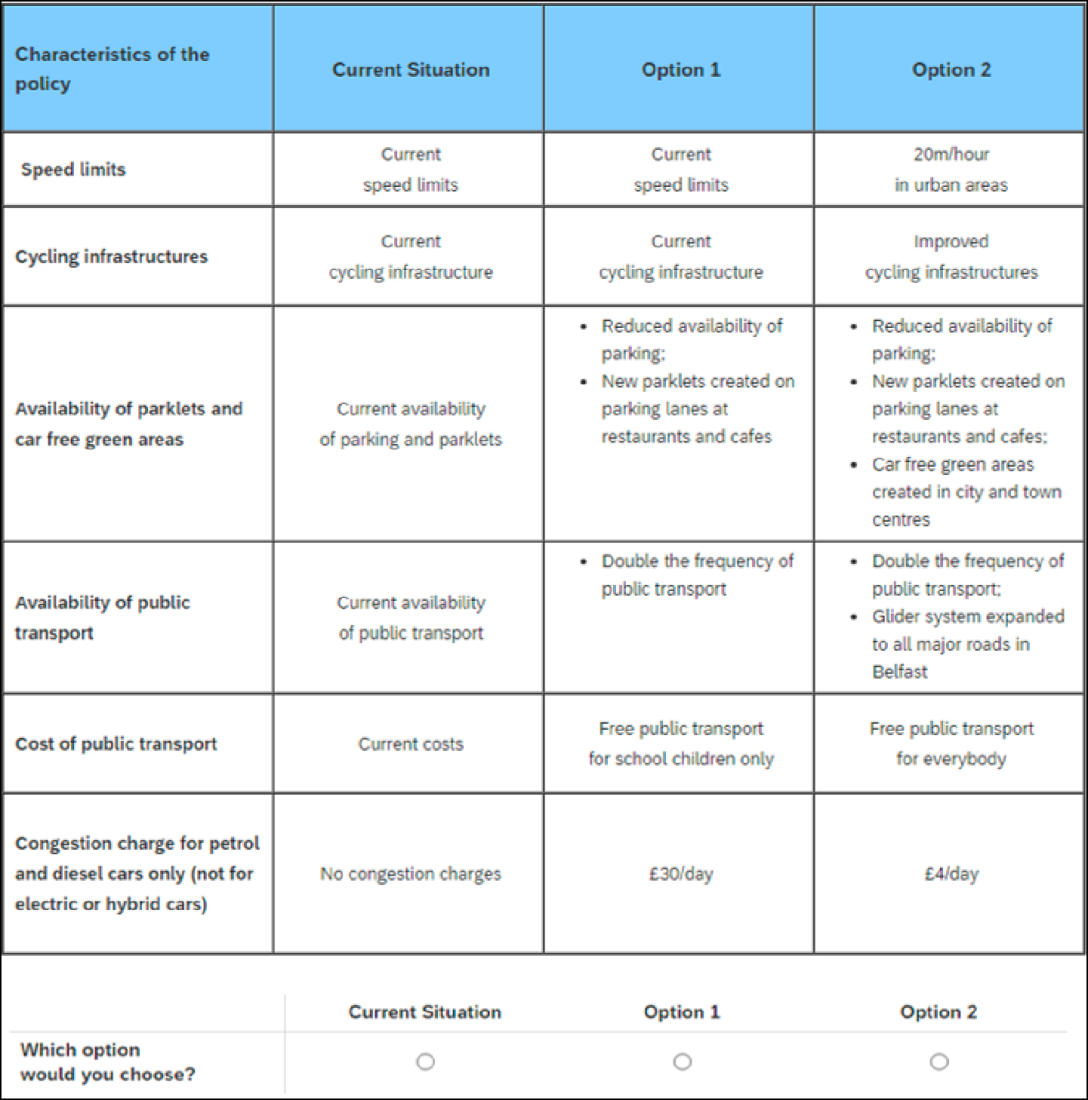
Example of a discrete choice experiment choice set

Figure 2 shows an example of a choice set included in the present study. Respondents were presented with 12 choice sets, each comprised of two experimentally designed alternatives and an opt-out alternative reflecting the current situation (or status quo). Each alternative was characterised by six attributes with varying levels [93].

The levels assigned to each attribute are shown in Table 1. Speed limits were defined as legally mandated speed limits enforced within all urban areas of Belfast. The levels of this attribute were: (i) the current speed limits of 30mph; and (ii) a decrease in the speed limit to 20mph. Cycling infrastructure was defined as provisions to enable people to cycle in and around the urban centre. This included facilities such as cycle lanes, greenways, bike rental stations, and bicycle storage. The levels of this attribute were: (i) the current availability of cycling infrastructure; and (ii) improved cycling infrastructure. Availability of parklets and car-free green areas was operationalised in two parts. First, parklets were defined as a sidewalk extension that provides more space and amenities for people using the street, at the cost of on-street parking spaces. Second, car-free green areas included any green space within the city that does not permit car access. The levels of this attribute were: (i) current availability of parking and parklets; (ii) reduced availability of on-street parking to accommodate parklets in all urban areas; (iii) reduced availability of on-street parking to create parklets, and the creation of car-free green areas in the city and town centres. Frequency and availability of public transport had the following levels: (i) current availability of public transport; (ii) frequency of public transport doubled; (iii) frequency of public transport doubled, and expanded availability of the rapid transit bus service; and (iv) frequency of public transport doubled, expanded availability of the rapid transit bus service, and connecting poorly served rural areas. Cost of public transport had the following levels: (i) current costs; (ii) free public transport for school children; and (iii) free public transport for everyone. WTP was assessed with a congestion charge (valid for one day) applied to petrol and diesel cars entering the city and had the following levels: (i) no charge; (ii) £0.25 per day; (iii) £1 per day; (iv) £4 per day; (v) £10 per day; and (vi) £30 per day.

**Table 1:** List of attributes and levels

Attribute	Current situation*	Level 1	Level 2	Level 3	Level 4	Level 5
Speed limits	Current speed limits	20mph in urban areas				
Cycling infrastructures	Current availability of cycling infrastructure	Improved cycling infrastructures				
Availability of parklets and car-free green areas	Current availability of parking and parklets	Reduced availability of on-street parking to create parklets on parking lanes at restaurants and cafes in all urban areas	Reduced availability of on street parking to create parklets on parking lanes at restaurants and cafes in all urban areas; creation of car-free green areas in city and town centres			
Frequency and availability of public transport	Current availability of public transport	Double the frequency of public transport	Double the frequency of public transport; Expanding the bus rapid transport system Glider to all major roads in Belfast	Double the frequency of public transport; Expanding the bus rapid transport system Glider to all major roads in Belfast; connecting poorly served rural areas		
Cost of public transport	Current costs	Free public transport for school children only	Free public transport for everybody			
Congestion charges in urban areas for petrol and diesel cars only	No congestion charges	£0.25/day	£1/day	£4/day	£10/day	£30/day

* Omitted during model estimation for identification purposes. Cost was assumed to be continuous

##### Experimental Design

The experimental design for the DCE was configured using an approach that was based on having obtained no prior information regarding the direction or magnitude of the parameters. Thus, it was assumed that the parameters (or priors) were all zero. To offset the risk of a less efficient design associated with setting the priors to zero, we assigned a relatively high number of choice tasks to each individual (i.e., 12) and ensured that the sample size was sufficient to enable reliable parameter estimation. The DCE was created in Ngene (https://www.choice-metrics.com/) using a $\:{D}_{Z}$-efficient [94] algorithm to construct a fractional factorial experimental design comprised of 60 choice tasks that were divided into five blocks of 12 choice tasks, and each respondent was randomly assigned to one block.

#### Contingent Valuation

**Fig. 3:**
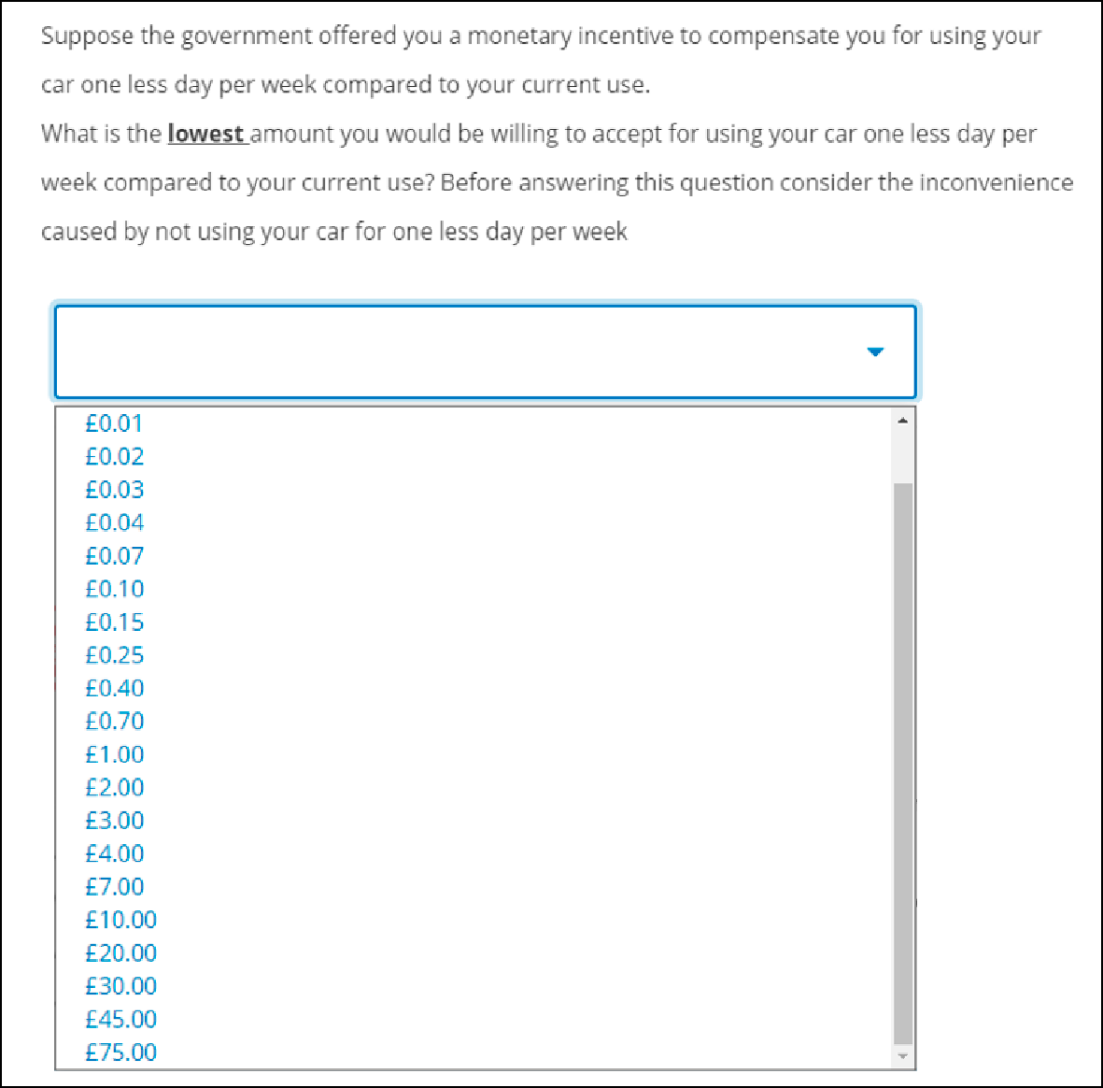
Contingent valuation question

WTA was captured using a payment ladder CV question (Fig. 3) where participants were asked to report the lowest amount they would be willing to accept from the government (in £) to compensate them for using their car one day less per week, with values ranging between £0.01 and £75.00. Following Rowe et al. [95] and Vossler et al. [96], the values offered to respondents in the payment card were chosen using an exponential response function of the form $\:{(1+k)}^{n-1}$ to generate a set of $\:n$ bids, where *k* was set equal to 0.6. The parameter 𝑘 controls the rate at which the bid amounts increase. We chose to have a long bid vector in order to adequately capture the distribution of WTA, and avoid problems associated with fat tails whereby a disproportionately high maximum bid can result in hypothetical bias [97, 98]. Further, setting the parameter *k* equal to 0.6 ensured that the bids did not escalate too quickly or slowly, while also considering the length of our bid vector and the initial bid value of £0.01, and balancing the need for a wide range of potential WTA values, thus ensuring that the increments between bids were manageable. The exponential response function also produced bids that are in line with the hypothesis that the accuracy at which respondents can estimate values is proportional to the value (i.e., it is easier to distinguish between a preference for £1 vs. £2 as opposed to £100 and £101) [96].

The final values used in the payment card were adjusted to avoid presenting abnormal values. For example, £29.51 was adjusted to £30. The exponential scale was used to mitigate against centering and range biases.

### Econometric Analysis

#### Discrete Choice Experiment - Preference Analysis

Based on random utility maximisation, it was assumed that respondents in the present study would make choices to maximise their utility [99, 100]. The utility ($\:{U}_{qjt}$) that respondent $\:q$ attaches to alternative $\:j$ is characterised by the vector of attributes, $\:{\chi\:}_{qkjt}$, in each choice set $\:t$. This can be expressed as follows:

1 $$\:{U}_{qjt}=\sum\:_{k=1}^{K}\left[{\beta\:}_{qk}{\chi\:}_{qkjt}\right]+{\epsilon\:}_{qjt}$$

where $\:{\beta\:}_{qk}$ is the random taste parameter of respondent $\:q$ for the attribute $\:k$; and $\:{\epsilon\:}_{qjt}$ is a random error term capturing the unmodelled part of the utility function that cannot be observed by researchers, and is assumed to follow a Gumbel distribution and be independent and identically distributed [101]. With these assumptions in mind, the probability of choosing a given alternative in each choice set can be described by a conditional multinomial logit (MNL) function:

2 $$\:\text{Pr}\left({Y}_{qjt}^{i}\right)=\frac{exp\:\left(\:\sum\:_{k=1}^{K}\left[{\beta\:}_{qk}{\chi\:}_{qkit}\right]\:\right)\:}{\sum\:_{j=1}^{J}exp\left(\:{\sum\:}_{k=1}^{K}\left[{\beta\:}_{qk}{\chi\:}_{qkjt}\right]\:\right)}$$

where $\:{Y}_{qjt}^{i}$ is the choice of alternative $\:i$ among $\:j$ alternatives by respondent $\:q$ for the choice set $\:t$. The MNL model assumes independence from irrelevant alternatives and homogeneity in preferences (i.e., each respondent has the same taste coefficient: $\:{\beta\:}_{qk}={\beta\:}_{k}$) [102]. The MNL specification was firstly used as a benchmark to explore the data, however, unobserved variation in preferences across the sample can bias estimates. Therefore, a mixed multinomial logit model (MXL) was deemed more appropriate. Mixed models are extensions of the conditional MNL model, and are commonly used in preference analysis to control for unexplained heterogeneity [103], as they estimate a distribution of preferences around each parameter that accounts for variations among individuals. Two types of MXL model specifications are commonly used: (i) one that assumes continuous distribution of preferences across respondents (e.g., random parameters); and (ii) one that assumes discrete distribution of preferences across respondents (e.g., latent class) [80, 104]. For this analysis, we used the random parameters logit (RPL) model specification.

By allowing the coefficients for each attribute level to vary across respondents [105, 106], the variance-covariance matrix of the RPL model can accommodate for: (a) independence of preference intensity or correlation (allowing for non-zero off-diagonal values); and (b) both preference and variance (or “scale”) heterogeneity [107]. In this study, normal distribution and independence of random parameters were assumed.

The estimated probability for choice $\:{Y}_{qjt}^{i}$ of alternative $\:i$ among $\:j$ alternatives by respondent $\:q$ represented below takes into account the panel nature of the data by incorporating the sequence of observed choices (T = 1…12):

3 $$\:\text{Pr}\left[{Y}_{qj1}^{i}\:,\dots\:,\:{Y}_{qjT}^{i}\right]=\:\int\:\prod\:_{t=1}^{T}\frac{exp\:\left(\sum\:_{k=1}^{K}\left[\left({\beta\:}_{k}+{\eta\:}_{k}\right){\chi\:}_{qkit}\right]\right)}{\sum\:_{j=1}^{J}\text{exp}\left(\sum\:_{k=1}^{K}\left[\left({\beta\:}_{k}+{\eta\:}_{k}\right){\chi\:}_{qkjt}\right]\right)}f\left({\eta\:}_{1.k}\right)d{\eta\:}_{1.k}$$

where $\:{\eta\:}_{k}$ is the random component that captures heterogeneity in the RPL model under the assumption of normally distributed random components – this represents the standard deviation of the distribution. The log likelihood function derived and maximised in our model estimation is therefore:

4 $$\:logL=\sum\:_{n=1}^{N}\text{ln}\int\:\prod\:_{t=1}^{T}\frac{exp\:\left(\sum\:_{k=1}^{K}\left[\left({\beta\:}_{k}+{\eta\:}_{k}\right){\chi\:}_{kit}\right]\right)}{\sum\:_{j=1}^{J}\text{exp}\left(\sum\:_{k=1}^{K}\left[\left({\beta\:}_{k}+{\eta\:}_{k}\right){\chi\:}_{kjt}\right]\right)}f\left({\eta\:}_{1.k}\right)d{\eta\:}_{1.k}$$

The integral in the equation above is approximated numerically using simulation methods [80] based on quasi-random Halton draws or modified hypercube sampling draws [108]. The maximised function is therefore called a simulated maximum log-likelihood function. For the present analysis, we used the maximum-likelihood simulation with 1,000 Halton draws.

The parameters for each attribute level presented in Table 1 were effects-coded and included as predictors in the regression model. An alternative specific constant was included for the status quo, and an error component was specified to nest the two experimentally-designed alternatives [109, 110]. Although dummy-coding and effects-coding are functionally equivalent, in this context, specifying each attribute level as effects-coded allowed for a direct interpretation of the alternative specific constant estimate [111]. In fact, effects-coding enables estimations of all levels (the omitted category can be retrieved as the negative sum of the others) and provides uncorrelated intercept estimates. Analyses were conducted in Stata 17.

#### Discrete Choice Experiment - Welfare Analysis

##### Marginal willingness-to-pay

The RPL model estimates were used to compute marginal WTP for policies that improved each attribute $\:k$ as the negative ratio between the utility improvement obtained by a policy that positively affected an attribute in the choice set and the marginal disutility of cost:

5 $$\:{WTP}_{k}=\:-\frac{{{\Delta\:}\beta\:}_{k}}{{\beta\:}_{cost}}$$

##### Compensating Variation

The compensating variation (also referred to as “consumer surplus”) [112–114] for specific policy scenarios was assessed by computing the difference in log-sum between the baseline scenario (current situation) and the policy change scenario, as described in the following equation:

6 $$\:{CV}_{n}=-\frac{1}{{\beta\:}_{price}}\left[\text{ln}{V}_{1}-\text{ln}{V}_{0}\right]$$

were $\:{CV}_{n}$ is the compensating variation for a change from initial conditions,$\:{V}_{0}$, to the conditions under the programme, $\:{V}_{1}$; and $\:{\beta\:}_{price}$ is the cost parameter which represents the marginal disutility of cost.

#### Contingent Valuation

To assess respondent’s minimum WTA a monetary incentive to reduce car usage, first, we created a double-bounded variable based on the lower and upper limits of the respondent’s WTA. The upper limit of WTA ($\:{B}_{q}^{u})$ was provided directly by the respondent’s answer. The lower limit of WTA ($\:{B}_{q}^{d}$) was assumed to be equal to the value below that of the respondent’s choice. For example, if the respondent selected £10.00, the lower limit was set to £7.00 as per the payment ladder shown in Fig. 3. The probability that a respondent chooses $\:{B}_{q}$ can be expressed as [115]:

7 $$\:\pi\:\left({B}_{q},{B}_{q}^{u}\right)=\text{Pr}\left\{{B}_{q}^{d}\le\:WTA\:\le\:{B}_{q}^{u}\right\}=G\left({B}_{q}^{u}|\theta\:\right)-G\left({B}_{q}^{d}\right|\theta\:)$$

Where $\:G\left({B}_{q}^{u}\right|\theta\:)$ is the cumulative density function. This yields the following log likelihood function:

8 $$\:logL=\sum\:_{i=1}^{n}\text{l}\text{o}\text{g}\left[\left({B}_{q}^{u}|\theta\:\right)-G\left({B}_{q}^{d}|\theta\:\right)\right]$$

As described in López-Feldman [116], we used maximum likelihood estimation to get estimates for $\:{\beta\:}_{q}$ that were used to estimate mean and median WTA. We then explored the impact of various factors on WTA by computing a multivariate interval data model.

## Results

Descriptive statistics are shown in Table 2. A large proportion of the sample were male (76%). The majority of respondents were between 18 and 64 years of age (87%). Most of the participants were skilled professionals, junior managers, or small business owners (37%), while a slightly smaller proportion indicated that they occupy senior or middle manager job roles, and specialised professional roles such as directors, doctors, and lawyers (30%). Household income fell between £30,000 and £99,000 for a majority of the sample (72%). Just over half (51%) of the sample had  ≥ 22 years of full-time education. This is consistent with the majority of participants who reported highest qualifications which were either university degree equivalent or higher (80%). With regard to nationality, just over a third of the sample (37%) indicated that they were either British only or Irish only. This is a very context-specific classification given Northern Ireland’s unique ethnopolitical context. Most households had two adults (67%), and no children (65%).

Over two-thirds of the sample were classified as overweight or obese (70%). The mean overall self-reported health score was 3.77 (SD: 0.91). It was therefore unsurprising that a low proportion of participants reported a long-term illness/disability or walking mobility problems (25% and 10%, respectively).

Just over half of respondents (53%) reported that poor availability of green spaces was a problem in the Belfast metropolitan area. Walking infrastructure was rated higher than public transport infrastructure with a mean score of 2.96 (SD: 1.12) compared to 2.84 (1.08), respectively.

**Table 2:** Sample characteristics

Sample characteristics^1^	Total (*n* = 773)	
Sex (male)	562	(76%)
Age, years		
18–44	290	(39%)
45–64	356	(48%)
≥ 65	97	(13%)
Occupation		
Unemployed/retired/student	166	(22%)
Community/voluntary Sector	17	(2%)
Clerk/tradesman/driver/labourer	59	(8%)
Junior manager/small business owner/skilled trade	278	(37%)
Senior or middle manager/director/doctor/lawyer	223	(30%)
Annual Household Income		
Less than £14,999	19	(3%)
£15,000 - £29,999	99	(14%)
£30,000 - £49,999	219	(32%)
£50,000 - £99,999	279	(40%)
More than £100,000	73	(11%)
Full-time education leaving age (in years)		
14–16 years	84	(11%)
16 years	9	(1%)
17–18 years	124	(17%)
19–21 years	147	(20%)
≥ 22 years	372	(51%)
Highest qualification		
No formal qualifications	11	(2%)
GSCE or A-level or equivalent	139	(19%)
Nursing, higher qualification or university first degree	363	(50%)
University higher degree	219	(30%)
Nationality		
Other	470	(63%)
Irish only or British only	273	(37%)
No. of adults in household		
1	114	(15%)
2	495	(67%)
3 or more	134	(18%)
No. of children in household		
0	485	(65%)
1–3	246	(33%)
> 3	12	(2%)
BMI		
Underweight/Healthy	204	(30%)
Overweight/Obese	483	(70%)
Health and Wellbeing		
Overall Health^2^	3.77	(0.91)
Long-Term Illness or Disability (Yes)	186	(25%)
Walking Mobility Problems (Yes)	77	(10%)
Poor availability of green space reported (Yes)	393	(53%)
Walking infrastructure rating^2^	2.96	(1.12)
Public transport infrastructure rating^2^	2.84	(1.08)

^1^ Variable distributions are reported as n (%) unless otherwise stated

^2^ Variable distributions are reported as mean (standard deviation)

### Discrete Choice Experiment Results

#### Marginal Willingness-to-Pay

The results from the RPL model are presented in Table 3. The table reports the coefficients, 95% confidence intervals, and p-values for the alternative-specific constant (status-quo), as well as each attribute and the standard deviations of each attribute. There was considerable improvement to the model fit of the data in the RPL model when compared to the MNL model (not reported), as indicated by an improvement in the log-likelihood function from − 4696.8 in the MNL model to -3155.8 in the RPL model.

The alternative specific constant representing the status quo option was negative, and statistically significant, indicating that respondents chose the status quo alternative significantly less than the other two alternatives. This suggests that respondents opted to pay a congestion charge for a hypothetical change in the attributes that comprised the choice sets, rather than maintain the status quo. The remaining estimated mean coefficients were positive with the exception of *doubled public transport frequency* and *free public transport for school children*, which were both non-significant at the 5% level.

**Table 3:** Results from RPL model

Name		Coefficient	95% confidence interval	*p*-value
*Alternative specific constant*				
	Status quo	-1.08	(-1.55–-0.61)	0.000
Attributes (means)*				
	20mph speed limits (baseline = 30mph)	0.01	(-0.06–0.09)	0.691
	Improved cycling infrastructure	0.29	(0.19–0.38)	0.000
	Reduced parking; new parklets	0.07	(-0.03–0.18)	0.173
	Reduced parking; new parklets; car free green areas	0.09	(-0.03–0.21)	0.138
	Doubled public transport frequency	-0.03	(-0.15–0.09)	0.610
	Doubled public transport frequency; expanded Glider	0.25	(0.12–0.38)	0.000
	Doubled public transport frequency; expanded Glider; connect poorly served rural areas	0.53	(0.38–0.67)	0.000
	Free public transport for school children	-0.10	(-0.20–0.00)	0.060
	Free public transport for everybody	0.35	(0.23–0.48)	0.000
	Congestion charge	-0.11	(-0.13–-0.09)	0.000
Attributes (standard deviations)*				
	20mph speed limits (baseline = 30mph)	-0.33	(-0.44–-0.22)	0.000
	Improved cycling infrastructure	0.67	(0.55–0.80)	0.000
	Reduced parking; new parklets	0.46	(0.30–0.61)	0.000
	Reduced parking; new parklets; car free green areas	-0.61	(-0.77–-0.45)	0.000
	Doubled public transport frequency	-0.34	(-0.59–-0.09)	0.008
	Doubled public transport frequency; expanded Glider	-0.37	(-0.60–-0.14)	0.001
	Doubled public transport frequency; expanded Glider; connect poorly served rural areas	-0.68	(-0.85–-0.51)	0.000
	Free public transport for school children	0.34	(0.16–0.52)	0.000
	Free public transport for everybody	-0.71	(-0.84–-0.58)	0.000
	Congestion charge	-0.12	(-0.15–-0.10)	0.000
Error component		-4.15	(-4.93–-3.38)	0.000
		**Value**		
Number of observations		13,914		
Number of respondents		773		
Log-likelihood		-3155.8		

*For each attribute, the omitted category can be retrieved as the negative sum of the estimated parameters

Table 4 shows the mean WTP (in £) as a daily congestion charge for the implementation of each attribute level as a change from the current situation.

**Table 4:** Mean WTP

Attribute	Attribute level	Mean WTP	Std. err.	z	*P*>|z|	95% CI
Speed limits	20mph in urban areas	£0.26	0.65	0.40	0.692	(-1.02–1.54)
Cycling infrastructures	Improved cycling infrastructure	£5.17	0.95	5.44	0.000	(3.30–7.03)
Availability of parklets and car-free green areas	Reduced parking; new parklets	£2.12	0.89	2.39	0.017	(0.38–3.86)
	Reduced parking; new parklets; car free green areas	£2.28	1.01	2.26	0.024	(0.31–4.26)
Frequency and availability of public transport	Doubled public transport frequency	£6.40	1.10	5.79	0.000	(4.23–8.56)
	Doubled public transport frequency; expanded Glider	£8.90	1.25	7.15	0.000	(6.46–11.35)
	Doubled public transport frequency; expanded Glider; connect poorly served rural areas	£11.43	1.43	7.98	0.000	(8.62–14.23)
Cost of public transport	Free public transport for school children	£1.39	0.91	1.53	0.126	(-0.39–3.18)
	Free public transport for everybody	£5.45	1.10	4.97	0.000	(3.30–7.60)

The results indicated that, on average, survey participants were willing to pay a daily congestion charge of £5.17 to fund improvements to cycling infrastructure. Respondents were willing to pay a daily congestion charge of £2.12 for the introduction of new parklets with a concomitant reduction in on-street parking. For a policy that also delivers car free green areas in city and town centres, in addition to parklets, respondents were willing to pay a £2.28 congestion charge. The highest level of congestion charge was supported for the provision of good public transport: £6.40 was the mean level of the congestion charge that respondents were willing to pay for doubling the frequency of public transport; if on top of doubling the frequency of public transport, the rapid transit bus service (“Glider”) is expanded, people would support a congestion charge of £8.90; if in addition to these measures, poorly connected rural areas of Northern Ireland are better connected with public transport infrastructure, the WTP a congestion charge increased to £11.43. Respondents were willing to see the introduction of a £5.45 daily congestion charge to support free public transport for everybody. Conversely, the results showed that there was not a statistically significant WTP to expand 20mph speed limits in urban areas beyond the city centre. Further, we did not observe a significant WTP to support the transition from the current provision of public transport infrastructure to offer free public transport for school children.

#### Compensating Variation Results

Table 5 shows the three policy scenarios (worst case, intermediate, best case) and compensating variation used assess the additional amount respondent were willing to pay for different combinations of the policy instruments. The results indicated that the aggregate WTP across respondents was £18.51, £25.45, and £35.13 for the combination of attributes and their corresponding levels that comprised the worst case, intermediate and best case scenarios, respectively.

**Table 5:** Policy scenarios and compensating variation

Attribute	Baseline	Worst case	Intermediate	Best case
Speed limits	Current speed limits	20mph in urban areas	20mph in urban areas	20mph in urban areas
Cycling infrastructure	Current availability of cycling infrastructure	Current availability of cycling infrastructure	Improved cycling infrastructures	Improved cycling infrastructures
Availability of parklets and car-free green areas	Current availability of parking and parklets	Reduced availability of on-street parking to create parklets on parking lanes at restaurants and cafes in all urban areas	Current availability of parking and parklets	Reduced availability of on street parking to create parklets on parking lanes at restaurants and cafes in all urban areas; creation of car-free green areas in city and town centres
Frequency and availability of public transport	Current availability of public transport	Double the frequency of public transport	Double the frequency of public transport; Expanding the bus rapid transport system Glider to all major roads in Belfast	Double the frequency of public transport; Expanding the bus rapid transport system Glider to all major roads in Belfast; connecting poorly served rural areas
Cost of public transport	Current costs	Current costs	Free public transport for school children only	Free public transport for everybody
**Compensating variation**	**N/A**	**£18.51**	**£25.45**	**£35.13**

### Contingent Valuation Results

The results of the CV question indicated that the mean WTA value reported in the survey was £14.38 (SD: 22.87). The median value, however, was £3.00 which is perhaps more practical from a policy perspective as it is not affected by the non-normal distribution of WTA. This suggests that the median respondent would need to be paid £3 each day they would be asked to give up using their car. In order for 75% of the sample to reduce car use by one day, the daily monetary incentive needs to rise to £20 (see Table 6).

**Table 6:** Summary statistics for willingness-to-accept

**Variable**	**Mean**	**S.D.**	Quantiles				
			**Min.**	**25%**	**50%**	**75%**	**Max.**
WTA	14.38	22.87	0.00	1.00	3.00	20.00	75.00

The results of the multivariate double-bounded dichotomous choice model for contingent valuation using maximum likelihood estimation are shown in Table 7.

**Table 7:** Results of interval data model

Variable	Coefficient	z	*p*-value	95%
Weekly car usage	0.99	3.86	0.000	(0.49–1.49)
Male	3.09	2.13	0.033	(0.24–5.94)
Age Group	-2.06	-1.99	0.046	(-4.09 - -0.03)
Occupation	0.15	0.28	0.777	(-0.86–1.16)
Income	-0.23	-0.31	0.758	(-1.72–1.25)
Education	-1.31	-1.93	0.053	(-2.63–0.02)
Qualification	-1.56	-1.62	0.105	(-3.45–0.33)
Nationality	1.25	0.98	0.328	(-1.26–3.77)
Household Adults	-2.23	-2.01	0.045	(-4.40 - -0.05)
Household Children	-0.91	-0.73	0.467	(-3.37–1.54)
BMI (Overweight)	1.25	0.90	0.367	(-1.47–3.97)
Health Score	1.32	1.51	0.131	(-0.39–3.04)
Illness/Disability	0.90	0.48	0.634	(-2.80–4.59)
Walking mobility	4.17	1.67	0.094	(-0.72–9.06)
Walk Infra. Rating	0.07	0.11	0.909	(-1.10–1.24)
Public Tra. Rating	-3.27	-5.44	0.000	(-4.45 - -2.10)
Green space avail.	-2.44	-1.93	0.053	(-4.91–0.03)
*Constant*	22.30	3.52	0.000	(9.90–34.71)

As the results show, weekly car usage was positively associated with WTA (β = 0.99, z = 3.86, *p* <.001). Of the sociodemographic factors, sex, age, education, and household composition were associated with WTA. Male sex was significantly positively associated with WTA (β = 3.09, z = 2.13, *p* =.033). In other words, males would require a larger monetary incentive to reduce their car usage by one day per week compared to females. Age was negatively associated with WTA (β = -2.06, z = -1.99, *p* =.046), suggesting that older car owners required less incentive to reduce their car usage compared to younger drivers. Occupation and income did not show a significant relationship with WTA, however, individuals with more years of education required less incentive to reduce their car usage (β = -1.31, z = -1.93, *p* =.053). Respondents living in households with a larger number of adults had a lower WTA (β = -2.23, z = -2.01, *p* <.045). Respondents who rated public transport infrastructure higher had a lower WTA (β = -3.27, z = -5.44, *p* <.001). Reporting poor availability of green spaces was negatively associated with WTA (β = -2.44, z = -1.93, *p* =.053).

### The Value of Reducing Car Usage in Belfast

The following sections describe the value of a policy intervention to reduce the number of weekly car journeys made by individuals living in Belfast with access to a car, focusing on the impact on greenhouse gas emissions, air pollution and road accidents only. Whilst other impacts such as noise, journey quality, commuting time, and physical activity may be relevant, these externalities would either be negative or have an uncertain sign, thus they would provide a more conservative estimate of the benefits of the hypothetical policy. The findings relate only to Belfast, and have not been extrapolated to another county or region in Northern Ireland or the UK.

#### Reduced Emissions and Air Pollution

Assuming that our sample is representative of drivers in Belfast, and knowing how many drivers there are, we calculated how much the government would have to pay to halve the number of cars on the road for one day per week. From this, we then calculated the value of the resultant reduction in carbon dioxide (CO_2_), particulate matter (PM), and nitrogen oxide (NO_X_) emissions.

The latest travel data that are available were published in March 2023 and reflect travel patterns and behaviours in 2021 in Northern Ireland [68]. On average, *n* = 583 journeys were made by car per person in 2021 (or *n* = 11.21 journeys per week). Not adjusting for seasonality, this equates to *n* = 1.60 journeys per person per day. The average journey length by car was 11.39 km. Thus, it was estimated that each person travelled 6646.59 km on average by car in Northern Ireland in 2021.

Mid-year population estimates for the Local Government District of Belfast which spans 133km^2^ published in August 2023 indicated that there were *n* = 271,409 people aged ≥ 18 years in 2021 [117]. In addition, census data indicated that 80% of households in Northern Ireland had access to a car or van [118], meaning that *n* = 217,127 people in Belfast aged ≥ 18 years have access to a car.

Assuming this portion of the population has access to a car and thus made on average *n* = 583 journeys in 2021 by car with an average length of 11.39 km, the total estimated number of journeys made by car was ~ 127 m, which equates to ~ 1.44bn kilometres travelled. As of June 2023, in the UK 59% of cars were petrol and 35% were diesel [119], therefore, it is estimated that ~ 851 m and ~ 505 m kilometres were travelled by petrol and diesel car, respectively (see Table 8).

At a cost of £3 per person applied to 50% of the population aged ≥ 18 years with access to a vehicle (*n* = 108,564) to reduce their car usage by one day per week, this policy would cost the government £325,692 per week, or £16,935,984 per year.

To produce a more conservative estimate of the value of a reduction in the number of car journeys by one day per week by half of the population aged ≥ 18 years with access to a car (*n* = 108,564) living in Belfast, we assumed each person made one journey per day that was on average 11.39 km. Therefore, a reduction in the number of car journeys made by one day per week (i.e., 52 across one year) would result in a reduction of 592.28 km travelled per person per year by car or 60.4 m kilometres for half of the population aged ≥ 18 years with access to a petrol or diesel car per year. For petrol car owners we estimate that this would be equivalent to 37.9 m fewer kilometres travelled and for diesel cars 22.5 m kilometres (see Table 8).

**Table 8:** Petrol and diesel car usage reduction in Belfast and policy costs

	Unit	
**Population aged ≥ 18 years**	n	271,409
**Proportion of households with access to a car**	%	80%
**Population aged ≥ 18 years with access to a car**	n	217,127
**Journeys made by car (per year)**	n	
*Petrol car (59% of Total)*		74,685,174.19
*Diesel car (35% of Total)*		44,304,764.35
*Total*		126,585,041
**Average journey length**	km	11.39
**Distance travelled by car (per year)**	km	
*Petrol car*		850,664,134
*Diesel car*		504,631,265.9
*Total*		1,441,803,617
**Cost of reducing car emissions**	£	
*Per week*		352,692
*Per year*		16,935,984
**Car journey reductions (per year)**		
50% of population aged ≥ 18 with car access	n	108,564
1 day less driving per week (per person)	km	592.28
1 day less driving (50% of population with car access)	km	64,299,989.78
*Petrol car*		37,936,993.97
*Diesel car*		22,504,996.42
Total*		60,441,990.39

* Calculated as the sum of petrol and diesel cars, excluding hybrid and electric vehicles

The calculations used to estimate the monetary value of reduced pollution attributable to a reduction in car use of one day per week by 50% of the population of Belfast with access to a petrol or diesel car are presented in Part B of the Appendix. Table 9 summarises the damage costs associated with each pollutant, as well as the value of reducing their corresponding emissions in line with the proposed policy instrument. The combined value of reduced emissions from CO_2_, PM, and NO_X_ is projected to be £2,845,050.

**Table 9:** Value of reduced emissions

Pollutant	Central damage cost (per ton)	Road transport source sector damage cost (per ton)	Value of estimated emissions (Belfast)	Value of reduced emissions (Belfast)
Carbon dioxide	£241	N/A	£54,296,392	£2,421,451
Particulate matter*				
*Exhaust*	£74,769	£84,548	£741,162	£33,054
*Non-exhaust*	£74,769	£84,548	£4,904,346	£218,719
Nitrogen oxide	£8,148	£11,682	£3,852,871	£171,826
		**Total**	**£63**,**794**,**771**	**£2**,**845**,**050**

* Includes both PM_10_ and PM_2.5_

#### Reduced Traffic-Related Injuries and Deaths

A report by the Department for Transport indicated that in 2022 there were *n* = 5 road fatalities per billion vehicle miles travelled in the UK [120]. Given that our proposed intervention is expected to hypothetically reduce petrol and diesel car usage by approximately 37,556,911 miles in its first year, we cannot conclude that there would any substantive impact on traffic-related fatalities in Belfast. However, such a reduction in miles travelled by car could conceivably reduce the number of traffic-related injuries, and thus, generate savings for the NHS through reduced admissions to hospital and emergency treatments. The same report indicates that in 2022 there were approximately *n* = 85 serious non-fatal casualties reported per billion vehicle miles travelled and *n* = 322 non-serious injuries.

Based on a reduction of 37,556,911 petrol and diesel car miles travelled as a consequence of the proposed intervention to reduce car usage by one day per week for 50% of the population with access to a car, we estimate that the number of serious non-fatal casualties will fall by *n* = 3 and the number of non-serious injuries by *n* = 12. It is estimated that the value of preventing an ambulance trip to a hospital accident and emergency (A&E) department in the UK is £17,148 (in 2022 prices) for serious casualties and £1,270 for non-serious casualties [121]. The value of preventing lost output attributed to serious and non-serious road casualties are £28,308 and £2,993, respectively. To capture the human costs associated with road casualties (i.e., the effects of injury, distress, pain, loss of life), an earlier study [122] measured individuals’ WTP for a marginal reduction in exposure to fatality risk, and found that the WTP (in 2022 prices) for serious and non-serious casualties is £204,890 and £14,998, respectively. The hypothesised reduction in petrol and diesel car usage will generate savings for the economy of £982,157 (see Table 10).

**Table 10:** NHS saving from reduced traffic-related injuries

Injury type	Ambulance and A&E visit cost	Lost output	Human costs	Proposed reduction	Savings
Serious non-fatal	£17,147.87	£28,307.60	£204,889.87	3	£751,036.02
Non-serious	£1,269.76	£2,992.71	£14,997.58	12	£231,120.60
				**Total**	**£982**,**156.62**

## Discussion and Conclusions

Using Belfast - a city with disproportionately high levels of congestion and car dependency - as a case study, we explored car owners’ WTP for a congestion charge that would fund hypothetical improvements to transport infrastructure and improve quality of life. Supplementary to the DCE, we asked car owners to report their WTA a monetary incentive to reduce their car usage by one day per week, and used this data to calculate the net benefit to the economy attributable to improved air quality and reduced road casualties. By employing two distinct survey-based techniques, we set out to generate complementary insights into improved urban transportation, and contribute to the growing body of literature that supports the role of market-based instruments, such as congestion charges and financial incentives, to reduce car use [123].

### The Congestion Charge as a Tool to Reduce Car Usage

The results of the DCE indicated that car owners were willing to pay a congestion charge between £2.12 and £11.43 for various policy measures, with the highest value attached to a policy that would improve public transport frequency and coverage, and expand rapid transit bus services. The compensating variation results showed that the aggregate WTP increased proportionately with the complexity and scope of hypothesised policy scenarios.

#### Perceptions and Attitudes

While previous studies have examined the acceptability of congestion charge schemes [124–126], as well as the difficulties associated with implementing them [127], our focus was on gauging car owners’ WTP for specific policy instruments using a congestion charge as the payment vehicle. It is possible that while respondents’ choices in the DCE reflected the congestion charge that they find acceptable, we cannot infer from the results that a congestion charge would alter their driving behaviour. However, we can conclude that approval of a congestion charge is likely tied to public perceptions around how the revenue generated through such a scheme will be utilised. For example, we observed that car owners are willing to incur the disutility associated with a congestion charge if access to car-free green areas in the city is improved, supporting findings from an earlier study by Grisolía et al. [124].

Congestion charge schemes need to be supported by a number of measures targeting drivers’ attitudes, namely: education about the environmentally damaging impacts of high car usage [128]; clear communication about the hypothecation of revenue from a congestion charge scheme [129]; transparency about how such funds will be created through fair and equitable market-based instruments [128]; and a “credible commitment” to use the funds for the purposes of achieving what was originally promised [130]. The results of the present study provide useful guidance for policymakers given that how they choose to publicise the introduction of a congestion charge scheme can have a significant impact on public support. Nevertheless, an understanding of car owners’ policy preferences and corresponding WTP is needed to establish acceptable limits for an appropriate congestion charge.

#### Potential Risks

The introduction of a congestion charge scheme in Belfast carries an element of risk. For instance, the existing public transport infrastructure might not be able to cope with the additional capacity pressure that a congestion charge scheme might create. However, this is not a certainty, as the revenue from the scheme could be used to fund more bus routes and improve service frequency. If the public perceive that public transport is insufficient, this could negatively impact on the acceptability of a proposed congestion charge [131].

There is also a risk that congestion charges will displace traffic as drivers attempt to circumvent charging zones [132]. This can be mitigated against by cordoning off the most congested areas of the city with a restrictive zonal scheme that charges drivers a daily toll for crossing the boundary to enter/exit a zone [131]. However, congestion zones need to be carefully partitioned, giving consideration to factors that precipitate congestion such as daily travel patterns [133], population density [134], vehicle ownership [135], land use patterns [136–138], as well as the spatiotemporal characteristics of traffic in the city [139, 140].

#### Supporting Sustainable Transport

Fostering sustainable transportation in cities is a complex endeavour. Simply creating new roads is not a viable solution, as this can induce greater demand (also referred to as the “Jevons Paradox” [141]) and result in increased car usage [142]. As such, cities like Belfast that are spatially constrained need policy solutions that support existing public transport and cycling infrastructure. A congestion charge can provide the additional funds needed to expand bus or rail services and improve cycling networks, and in so doing increase the modal share of sustainable transport alternatives by providing commuters with an efficient substitute to driving. A stronger public transit system might make car owners more amenable to a congestion charge if it means switching transportation mode will not impose an excessive time penalty [143].

#### Directions for Future Research

While the findings from this study offer insight into how car owners’ WTP for a congestion charge varies conditional on the characteristics of specific policy instruments, there is scope for further research in this area. For example, we did not explore how WTP varied across owners of different vehicle types or how age of the vehicle may have impacted WTP. It is possible that owners of newer vehicles with lower emissions would be willing to pay less given the financial outlay associated with purchasing a newer car. However, creating exemptions for cleaner vehicles risks undermining the success of such programmes, and can further exacerbate inequalities if wealthier car owners are better off. There are also equity considerations that need to be explored in future research. For instance, there is a risk that a congestion charge scheme based on vehicle type or emissions could disproportionately penalise owners of older, more heavily polluting vehicles which typically tend to be low-income drivers who cannot afford to buy a newer car.

### Financial Incentives as a Tool to Reduce Car Usage

In addition to the DCE, we used a payment ladder CV question to elicit respondents’ WTA a monetary incentive to reduce their car usage. The results indicated that 50% of car users were willing to accept £3 as minimum compensation for using their car one day less per week, and that 25% would do the same if they were offered £1. This value increased to £20 for 75% of the sample, which is substantially higher and underscores how expensive it would be to incentivise car owners to forfeit their driving privileges, even for one day a week. For comparison, the value of an adult day ticket for Translink Metro and Glider bus services within the city is £5.00 (https://www.translink.co.uk/).

#### Factors Associated with Willingness-to-Accept a Monetary Incentive to Reduce Car Use

When assessing the factors that contributed to WTA, we found that, unsurprisingly, respondents that used their car more frequently had a higher WTA. We also found that males required a larger monetary incentive to reduce their car usage. Despite earlier studies finding that males used private transportation modes such as cars more frequently than females [144–146] - which could explain a higher WTA - petrol and diesel car usage did not vary significantly across the two groups in the present study. Consequently, the disparity in WTA across males and females could in part be explained by attitudinal factors related to car use [146] or social factors [147].

We also observed that older participants had a lower WTA, mainly accounted for by those who were aged ≥ 65 years. A large proportion of this group were classified as unemployed or retired – two groups that used their car less frequently. This trend coincides with a previous study that reported people aged ≥ 65 years travelled less frequently than their younger counterparts [145]. Moreover, the lower WTA can also be attributed to people aged ≥ 60 years in Northern Ireland being entitled to free public transport, which reduces the opportunity cost of foregoing the right to drive for one day per week.

Survey participants who had more years of education had a lower WTA, suggesting that individuals with fewer years of education occupy job roles that necessitate more driving, which is reflected in a higher WTA. Those whose job classification was Clerk/Tradesman/Driver/Labourer had the highest weekly car usage and the fewest years of education. We also observed that weekly car usage was highest among the subgroup with no formal qualifications and lower among those with university or higher degrees. Given the strength of the relationship between weekly car usage and WTA, we can infer that the relationship between education and WTA is largely driven by work-related factors rather than education per se. This is broadly in line with the results of an earlier study that found individuals with higher levels of education were less likely to drive a car and used public transport more frequently [148]. We observed a similar pattern whereby individuals with higher qualifications used public transport more frequently, as well as active transport modalities such as walking and cycling (not reported).

#### Calculating the Benefits of Reduced Car Usage

We estimated that to reduce the number of journeys made by car by one day per week among half of the population with access to a car in Northern Ireland would cost the government £16.9 m per year. As a result of reduced emissions and road casualties, it was estimated that this intervention would generate benefits to society worth £3.83 m, which were far outweighed by the cost of the intervention (with a cost-benefit ratio of 4:1). Supplementary analysis (not reported) indicated that if 25% of drivers accepted a £1 monetary incentive to reduce their car usage by one day per week at a cost of £2.82 m, the estimated annual benefits would be worth £1.42 m. As such, even a small reduction in car usage attributable to the proposed policy instrument would not pass a cost-benefit analysis given the cost-benefit ratio of 2:1. This does not preclude the possibility that interventions such as this are financially viable. However, this does suggest that the implementation of such a programme will largely be contingent on a government’s ability to absorb the financial expenditure to fund and justify it.

It is worth highlighting that the present analysis focused primarily on air pollution, greenhouse gas emissions, and traffic-related accidents. We did not account for other externalities such as reduced commuting time or improved journey quality. Other factors, such as noise and physical activity, are more ambiguous. For instance, reduced traffic might lead to higher speeds which in turn can result in higher noise levels due to tyre-road friction. Alternatively, less traffic might lead to a decrease in noise levels due to reduced acceleration, deceleration, and horn use. Similarly, physical activity could increase if people continue commuting, or decrease if remote work becomes more prevalent as a result of the need to avoid congestion charges. Nevertheless, factoring in these potential outcomes will likely do little to change the result that the cost of reducing car usage by one day per week exceeds the associated benefits by more than four times.

### Conclusions

Based on the results of the DCE and the payment ladder CV question, we have drawn three main conclusions: (i) car owners have clear preferences for specific transport policies as they are linked to a congestion charge, and WTP varies as a function of the combination of attributes and their respective levels that make up each policy scenario with WTP increasing proportionately with more comprehensive policy configurations; (ii) the congestion charge identified for the intermediate policy scenario is comparable with other cities; and (iii) the cost of introducing a £3 monetary incentive to reduce car usage by one day per week among 50% of the population with access to a car would outweigh the potential benefits by a factor of four, making it difficult to justify the implementation of such a measure to reduce car usage.

## Electronic Supplementary Material

Below is the link to the electronic supplementary material.

Supplementary Material 1

## Data Availability

Data will be made available on reasonable request to the study team.

## Abbreviations

CV: Contingent valuation
DCE: Discrete choice experiment
gNO_X_e: Equivalent gram of nitrogen oxide
kgCO_2_e: Equivalent kilogram of carbon dioxide
mgPMe: Equivalent milligram of particulate matter
MNL: Multinomial logit
MXL: Mixed logit
NO_X_: Nitrogen oxide
NPV: Net present value
PM: Particulate matter
RPL: Random parameters logit
tCO_2_e: Equivalent ton of carbon dioxide
tNO_X_e: Equivalent ton of nitrogen oxide
tPMe: Equivalent ton of particulate matter
UK: United Kingdom
WTA: Willingness to accept
WTP: Willingness to pay
