# Supplementary material for: A Stated Preference Study to Explore Market-Based Instruments to Reduce Car Usage
Source: Environ Resour Econ (Dordr). 2025 Jun 5;88(8):2201–33. doi: 10.1007/s10640-025-01005-w (PMC12263728; doi:10.1007/s10640-025-01005-w)
Supplement: Supplementary file 1 — Supplementary Material 1 [file 10640_2025_1005_MOESM1_ESM.docx]

**Appendix**

Contents

[Part A – Study design, limitations, and policy context 3](#_Toc190161856)

[A.1 Study sampling method 3](#_Toc190161857)

[A.2 Survey instruments 3](#_Toc190161858)

[A.2.1 Car usage, access to green space, walking infrastructure rating, and public transport infrastructure rating 3](#_Toc190161859)

[A.2.2 Sociodemographic and health characteristics 4](#_Toc190161860)

[A.3 Study Limitations 9](#_Toc190161861)

[A.4 Policy context 10](#_Toc190161862)

[A.4.1 Overview 10](#_Toc190161863)

[A.4.2 The Regional Development Strategy for Northern Ireland 11](#_Toc190161864)

[A.4.3 Strategic Planning Policy Statement 12](#_Toc190161865)

[A.4.4 Regional transport policy 13](#_Toc190161866)

[A.4.5 Investment in public transport 13](#_Toc190161867)

[A.4.6 Investment in active transport 15](#_Toc190161868)

[Part B – Car emissions 16](#_Toc190161869)

[B.1 Greenhouse gas emissions 16](#_Toc190161870)

[B.2 Particulate matter emissions 18](#_Toc190161871)

[B.2.1 Exhaust emissions 18](#_Toc190161872)

[B.2.2 Non-exhaust emissions 21](#_Toc190161873)

[B.3 Nitrogen oxide emissions 25](#_Toc190161874)

[References 27](#_Toc190161875)

List of Tables

[Table S1: Average CO_2_ emissions (per car) 16](#_Toc190161876)

[Table S2: Value of CO_2_ emissions 16](#_Toc190161877)

[Table S3: Average exhaust particulate matter emissions (per car) 18](#_Toc190161878)

[Table S4: Value of exhaust particulate matter emissions 18](#_Toc190161879)

[Table S5: Emission factors for PM_10_ from non-exhaust emissions (mg PM_10_/km) 21](#_Toc190161880)

[Table S6: Fraction of PM10 emitted as PM2.5 for non-exhaust traffic emissions 21](#_Toc190161881)

[Table S7: Average non-exhaust particulate matter emissions (per car) 22](#_Toc190161882)

[Table S8: Value of non-exhaust particulate matter emissions 22](#_Toc190161883)

[Table S9: Average NO_X_ emissions (per car) 25](#_Toc190161884)

[Table S10: Value of exhaust NO_x_ emissions 25](#_Toc190161885)

# Part A – Study design, limitations, and policy context

## A.1 Study sampling method

For the present study, the survey targeted a subset (n=2,200) of the established Northern Ireland LucidTalk online Opinion Panel (n=14,517 members). Individuals within this subset who indicated that they owned a car were invited to participate in the poll-project

LucidTalk (LT) run scheduled regular 'Tracker' polls of their established Northern Ireland (NI) online Opinion Panel - usually on a quarterly basis. The online LT Opinion Panel (n=14,517 members) consists of NI residents (age 18+) and is balanced by sex, age-group, area of residence, and community background, in order to be demographically representative of NI. The objective of these scheduled and regular 'Tracker' polls is to research opinion on key issues at key points in time and to track changes in trends/opinions about key topics and issues over specific periods of time. Usually a LT 'Tracker' poll-project targets a specific sector (designed by computer) of the 14,517 member online NI Opinion Panel, and invite this sub-set of the LT NI Opinion Panel to participate, and a set number of responses will be received – usually 3,000-4,000 responses. All responses then undergo a data auditing process to ensure:

- 'one person-one vote' responses; and
- the collation of a robust, accurate, and balanced representative NI sample.

The project was carried out online in the time-period from 18th to 25th February 2022. The project targeted the established NI LT online Opinion Panel. 773 full responses were received, and a data auditing process was then carried out to ensure all completed poll-survey responses are/were genuine 'one-person, one-vote' responses, and also to collate a robust and accurate balanced NI representative sample.

## A.2 Survey instruments

### A.2.1 Car usage, access to green space, walking infrastructure rating, and public transport infrastructure rating

Weekly petrol/diesel car usage was assessed with a single item (“When travelling, how often do you use a petrol or diesel car?”) measured on a nine-point scale from 1 = “Never” to 9 = “Seven days per week”.

Self-reported access to green space was assessed with a single item (“Do you think that the following are important problems in the Belfast metropolitan area… Poor availability of green spaces?”) reported on a dichotomous scale (0 = “No” and 1 = “Yes”).

Participants were asked to rate the existing walking infrastructure with a single item (“How would you rate walking infrastructure, such as the quality and safety footpaths, availability of benches, resting areas, and green space where you live?”) and public transport infrastructure with a single item (“How would you rate public transport where you live?”). Both were measured on a five-point scale from 1 = “Very poor” to 5 = “Very good”.

### A.2.2 Sociodemographic and health characteristics

The survey instruments used to capture the respondents’ sociodemographic characteristics are shown below:

Please state your sex.

|  | Please tick |
| --- | --- |
| Male | 🞏 |
| Female | 🞏 |
| Other | 🞏 |

Please state your age (in years).

|  | Please tick |
| --- | --- |
| 18 - 24 | 🞏 |
| 25 - 34 | 🞏 |
| 35 - 44 | 🞏 |
| 45 - 64 | 🞏 |
| 65 + | 🞏 |

What is your occupation?

|  | Please tick |
| --- | --- |
| Unemployed/retired/full-time education | 🞏 |
| Community/Voluntary Sector worker | 🞏 |
| Clerk/Tradesman/Driver/Labourer | 🞏 |
| Junior Manager/Small Business owner/Skilled Trade/Engineer/Teacher | 🞏 |
| Senior or Middle Manager/Co. Director/Doctor/Lawyer | 🞏 |

What is your household income (per year)

|  | Please tick |
| --- | --- |
| under £5,000 | 🞏 |
| £5,000 - £14,999 | 🞏 |
| £15,000 - £24,999 | 🞏 |
| £25,000 - £34,999 | 🞏 |
| £35,000 - £49,999 | 🞏 |
| £50,000 - £69,999 | 🞏 |
| £70,000 - £99,999 | 🞏 |
| £100,000 - £149,999 | 🞏 |
| £150,000 + | 🞏 |

Please state your highest educational qualification.

|  | Please tick |
| --- | --- |
| No formal qualifications | 🞏 |
| GCSE or A-level or equivalent | 🞏 |
| Nursing, technical, professional, or higher qualification | 🞏 |
| University first degree | 🞏 |
| University higher degree | 🞏 |

Please state what age you were when you left school.

|  | Please tick |
| --- | --- |
| 14 – 15 years | 🞏 |
| 16 years | 🞏 |
| 17 – 18 years | 🞏 |
| 19 – 21 years | 🞏 |
| 22 + years | 🞏 |

Please state your nationality.

|  | Please tick |
| --- | --- |
| Irish only or British Only | 🞏 |
| Other | 🞏 |

Please state how many adults live in your household.

|  | Please tick |
| --- | --- |
| One | 🞏 |
| Two | 🞏 |
| Three or more | 🞏 |

Please state how many children live in your household.

|  | Please tick |
| --- | --- |
| None | 🞏 |
| One | 🞏 |
| Two | 🞏 |
| Three or more | 🞏 |

Health and wellbeing was assessed with four questions. First, respondents were asked to rate their overall health with a single item (“In general, how would you rate your overall health?”) on a five-point scale from 1 = “Very poor” to 5 = “Very good”. Second, long-term illness or disability was measured with a single item (“Do you have any long-term illness, health problem or disability which limits your daily activities or the work you can do? Include problems which are due to old age.”) measured on a dichotomous scale (0 = “No” and 1 = “Yes”). Third, walking mobility was measured with a single item (“Do you have any difficulty walking for a quarter of a mile (about 400 metres) on a level surface?”) measured on a dichotomous scale (0 = “No” and 1 = “Yes”). Finally, body mass index (BMI) was derived from participants’ self-reported height and weight, and was coded as a categorical variable with participants being classified as either underweight/healthy (BMI ≤ 24.9) or overweight/obese (BMI ≥ 25).

It is worth noting that the sample was comprised of car owners and, therefore does not necessarily reflect the sociodemographic profile of Belfast as a whole. Nevertheless, there are comparisons to be drawn between the two groups.

Given the sunk cost of purchasing a car, and the costs associated with running a car (i.e., fuel, servicing, maintenance, insurance, road tax), it was expected that the sample would have a higher income, and thus more likely to be employed and better educated compared to the general population. This is reflected in the relative preponderance of respondents in the 45-64 age group when compared to the general population of Belfast – an age group that, in the present study, had a larger proportion of respondents in high-earning senior job roles, which in turn generate a higher household income and require employees to be better educated.

The table below compares the age profile of the study sample (car owners) and the population of Belfast based on 2022 mid-year population estimates published by the Northern Ireland Statistics and Research Agency (NISRA) in August 2023 (1).

| **Age group** | **Current study** | **Belfast** |
| --- | --- | --- |
| 18-44 | 39% | 51% |
| 45-64 | 48% | 30% |
| 65+ | 13% | 19% |

Income was captured at the household level and may not in every instance represent individual-level income in the present study. We observed that a large proportion of the sample (66%) lived in households with two adults, resulting in a high average household income. The most recent employee earnings report published by NISRA in November 2023 (2) indicated that the mean annual gross pay for an individual living in Belfast was £36,371. As such, it was not surprising to find that the majority of respondents in the present study reported a household income of £30,000 - £49,999 (32%) and £50,000-£99,000 (40%).

With regard to household composition and the number of children in each household, the sample did not differ significantly from the general population of Belfast, with the exception of a slightly higher proportion of respondents with no children. The table below shows household composition data that were obtained from the 2021 Census (3) compared with the sample from the current study.

| **Number of children** | **Current study** | **Belfast** |
| --- | --- | --- |
| 0 | 65% | 56%* |
| 1 | 14% | 15% |
| 2 or more | 21% | 29% |
| * Includes: households with non-dependent children aged ≥ 18 years; one person households; single family households (all aged 66 and over); single family household (married or civil partnership); single family household (co-habiting couple); single family household (other family composition); all in full-time education (students); other household types (all aged 66 and over); and other household types (other family composition). | | |

Regarding long-term illnesses and disabilities, Census 2021 data reported by NISRA indicated that that 26.7% of people living in Belfast have a limiting long-term illness or disability (<https://www.nisra.gov.uk/system/files/statistics/census-2021-main-statistics-for-northern-ireland-phase-2-statistical-bulletin-health-disability-and-unpaid-care.pdf>). This aligns closely with the figure reported by respondents in the current study (i.e., 25%). The same document indicated that 10% of the population of Belfast reported ‘bad or very bad’ general health. This is comparable with the proportion of respondents in the current study that reported ‘poor or very poor’ overall health (8%).

Data published by the Department of Health (<https://www.health-ni.gov.uk/news/health-survey-ni-first-results-201920>) in December 2020 indicated that in Northern Ireland 65% of adults were classified as either overweight or obese, with males more likely to be overweight. By comparison, in the present study, 70% of respondents were classified as either overweight or obese. The sample was also made up of a large portion of males (i.e., 76%) which could explain the higher proportion of individuals classified as overweight and obese.

## A.3 Study Limitations

There were a number of limitations associated with the current study. First, as the data were cross-sectional, the results provided only a snapshot of car owners’ WTP for a congestion charge. It is possible that car owners’ attitudes and preferences change over time in conjunction with other social, political, and economic developments, but that was beyond the scope of this study to control for. Second, there are limitations inherent in using stated preference methods and more specifically DCEs to elicit WTP. These include hypothetical bias (4) and embedding effects (5). To mitigate against hypothetical bias, a number of steps were taken, namely: i) we provided an opt-out alternative in each choice set to ensure that respondents were not forced to select an alternative with a payment (6); ii) we used a choice-based elicitation mechanism to gather each respondent’s relative valuation for each combination of attributes as this most accurately reflects decision-making scenarios that individuals are tasked to make in a real-world context; and iii) we incorporated “cheap talk” into the survey design (7,8) to assure participants that there were no right or wrong answers, to remind them of budget constraints, and to inform them that the choice sets are independent and not ordered – this also helped to reduce order effects. Third, previous studies have demonstrated that income, something that was not controlled for in the current study, can impact on WTP and in scenarios where respondents are bound by income constraints, the maximum amount people are willing to pay can be affected. Future research in this area could explore how WTP for a congestion charge differs across different subgroups stratified according to income.

Another limitation of the present study relates to the calculations presented in Section 3.3 to estimate the value of reduced car usage in Belfast. The calculations are based on reducing the number of car journeys made per week by half of the population in Belfast aged ≥ 18 years with access to a car, rather than people aged ≥ 18 years who own a car. We have therefore conflated those who own a car and those have access to a car. It is plausible that those who have access to a car that they do not own would require a lower monetary incentive to reduce their car usage as they have not incurred the sunk cost of having purchased the vehicle. Thus, it is likely that we have overestimated the cost of a programme to reduce car usage by one day per week in Belfast by assuming the median willingness-to-accept value (£3.00) would be the same across both car owners and those who only have access to car but do not own it. Consequently, the net benefit to the economy that we have calculated from such a programme is arguably conservative, as it assumes that the cost to reduce car usage by one day per week does not vary across the two groups. Another factor contributing to a potential underestimation of the net benefits of such an intervention relates to travel times. We did not attempt to model a potential reduction in travel times associated with a reduction of car journeys made by half of the population with access to a car. We acknowledge that congestion, which results in increased travel times, incurs a social cost to society, but data regarding travel times for car journeys in Northern Ireland were not readily available. This is something that should be explored in future research as it could have a positive impact on the net value of interventions to reduce car usage.

## A.4 Policy context

### A.4.1 Overview

The policy context for transportation in Belfast combines elements from strategic and local planning policy, urban regeneration frameworks, as well as housing and neighbourhood development strategies (9). Figure 1 shows the overarching transport policy context set by the Regional Development Strategy (RDS) 2035 (10); the Strategic Planning Policy Statement (SPPS) (11) (specifically Planning Policy Statements 3 (12) and 13 (13)), and the Development Control Advice Note 15 (14) which covers detailed aspects of siting, design, visibility and safety of road layouts on new developments. The policy document titled “Ensuring a Sustainable Transport Future: A New Approach to Regional Transportation” (15) relates to the Regional Development Strategy 2035 and also provides a context for the Accessible Transport Strategy 2025 (16), as well as specific transport plans and studies that underpin the new Local Development Plans (LDPs). The LDPs are in various stages of preparation by the 11 post-2015 Council areas that followed the Reform of Public Administration in Northern Ireland.

Figure 1: Policy context for transport in Northern Ireland


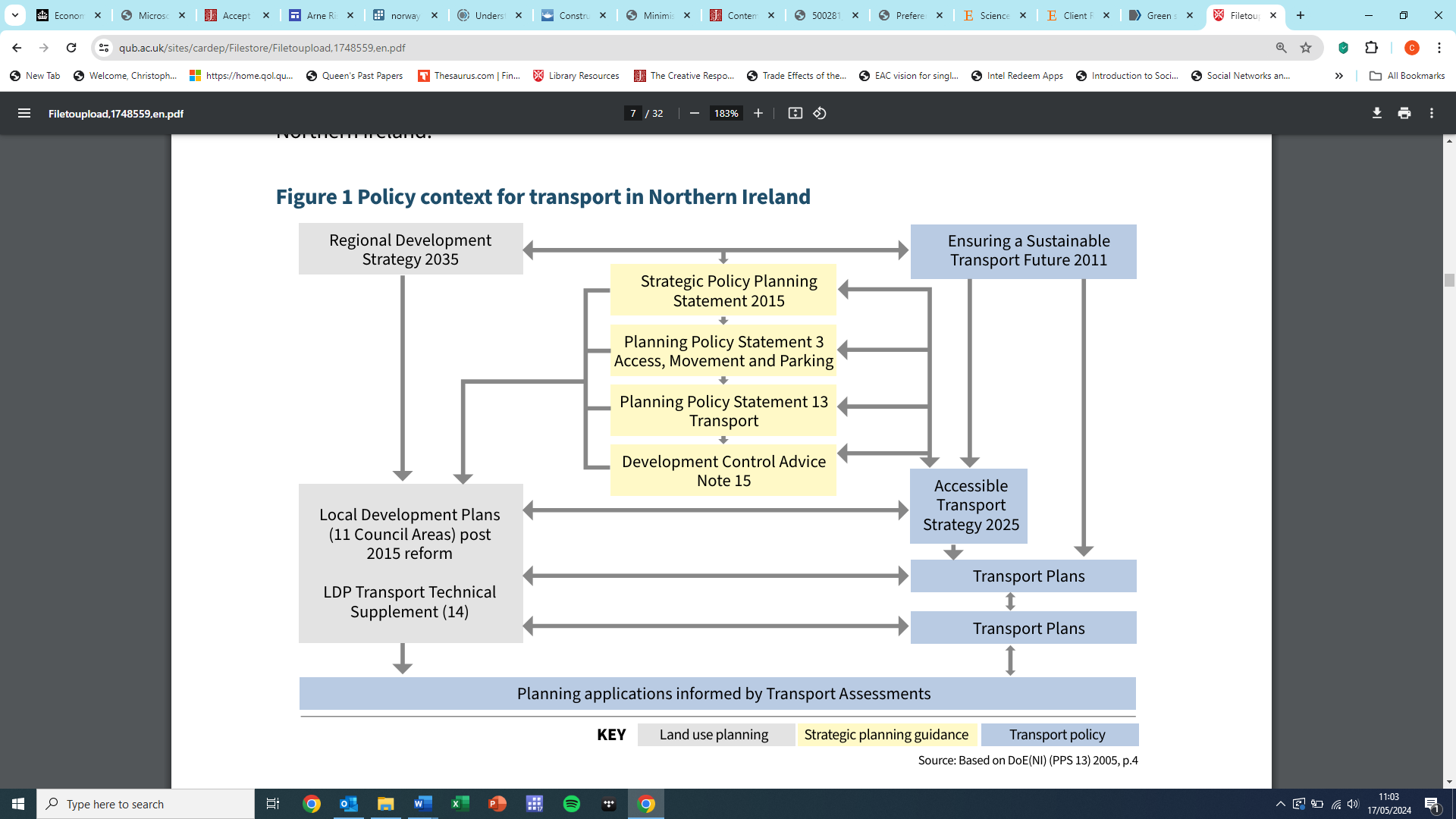


*Source: adapted from Department of the Environment (NI) (2005) Planning Policy Statement 13: Transportation and Land Use, Belfast: The Planning Service, p.4*

### A.4.2 The Regional Development Strategy for Northern Ireland

The RDS (and related strategies) aims to support a modal shift away from the car through a number of mechanisms beyond transport, namely: encouragement of compact neighbourhoods; environmental sustainability; and the development of land within the existing urban footprint to mitigate against urban sprawl, thereby reducing the burden of extended commuting (9). The RDS also stresses the importance of promoting climate resilience and creating a city that can adapt to climate change by exploring potential synergies across transport and energy planning, and how these intersect with population living arrangements and work patterns. The RDS also highlights the need to reduce reliance on carbon-based transport modes through increased opportunities for walking, cycling and public transport, which will in turn improve air quality by reducing greenhouse gas emissions (10).

The Spatial Framework Guidance 2 (SFG2: Grow the population of the City of Belfast) contained within the RDS (10) states that a balanced approach to transport infrastructure is needed for economic growth, stating that ‘to remain competitive in the global market it is important to continue to promote transport which balances the needs of our environment, society and economy’ (p.35). It sets out a new approach to transport in that ‘we need to maximise the productivity of our transportation network, particularly the use of well-maintained finite amount of road space. The capacity of road space can be increased by moving people in a more efficient way and by providing more attractive transport choices’ (p.35). Increasing the use of road space will, the strategy acknowledges, require an improvement in the public transport service (p.35):

*“Continued investment in public transport and in infrastructure such as the development of quality multi-modal facilities and park and ride sites, will encourage motorists to take the bus or train for the main part of their journey and reduce the volume of traffic on the network*.”

The strategy also acknowledges that such an approach will help to deliver better social inclusion outcomes (p.35):

*“This will mean seeking innovative public transport services including transport programmes focused on the user and services that meet the needs of communities. These will include Door-to-Door services, demand responsive services, Rapid Transit and services tailored to the needs of older people and people with disabilities*.”

### A.4.3 Strategic Planning Policy Statement

The SPPS for Northern Ireland (11) sets out regional guidance for delivery of better integrated land use and transport at local authority level. This includes improvements to connectivity and the development of sustainable transport infrastructure, and draws a clear link between better public health and transport. Planning Policy Statement (PPS) 13 (Transport and Land Use now in the SPPS) was especially important in guiding the implementation of the RDS and directly challenges the regions’ traditional reliance on the car and its socio-environmental impacts (p.8):

*“Whilst the car brings advantages in terms of personal mobility, its use contributes to environmental problems in our cities and towns. Also, an emphasis on the car in the planning of development increases car dependency as well as influencing the built form and layout of urban areas.”*

The SPPS (11) also outlines specific regional strategic objectives for transportation and how they relates to land-use planning (pp.106-107):

- Promote sustainable patterns of development which reduce the need for motorised transport, encourages active travel, and facilitate travel by public transport in preference to the private car;
- Promote the provision of adequate facilities for cyclists in new development; and
- Promote parking policies that will assist in reducing reliance on the private car and help tackle growing congestion.

### A.4.4 Regional transport policy

The first Regional Transportation Strategy (RTS) for Northern Ireland (17) emphasised the poor performance of transport and a sustained reduction in walking and cycling; increase in car use and especially for trips to school; and declining public transport in which Citybus journeys fell from 25.4m journeys in 1995/6 to 20.3m in 2001 (p.35). The RTS was mapped on to the Spatial Development Strategy set out in the first RDS (18) and in particular emphasised the importance of connectivity within and between Belfast and other towns, the island overall and with Great Britain. The strategy aimed to spend £3,500m on transportation of which £2,181.1m (63%) was allocated to roads and £1,232.1m (35%) to public transport but as is shown, despite a considerable impact, this balance in investment has not been maintained.

### A.4.5 Investment in public transport

Progress has been made in infrastructure investment relating to public transport, walking, and cycling. As previously alluded to, the Regional Transport Strategy (17) included an investment of £3.5bn in bus fleets, service routes, infrastructure, and railway rolling stock. This helped to successfully shift the modal balance in a relatively short period of time. The strategy indicated that £1.23bn (35%) was to be spent between 2002 and 2012 on public transport and £2.18bn (63%) on roads.

Figure 2 shows the change in public transport performance over the life of this strategy and the progress that was made after 2012 (since the new regional transport plan was adopted). The figure shows a clear increase in the number of journeys and receipts across bus and rail networks, as well as an improvement in service provision (captured in number of buses and staff) that were sustained after 2012. The age of the bus fleet continued to decline past 2012, which was in part aided by the introduction of the rapid transit bus (Glider) service. Performance continued to improve after the first RTS, but at a much slower rate. Receipts were one-quarter of the growth in the last decade compared with the 10-years before, and growth in the number of passenger journeys across bus and rail networks slowed substantially.

Figure 2: Public transport and strategic planning


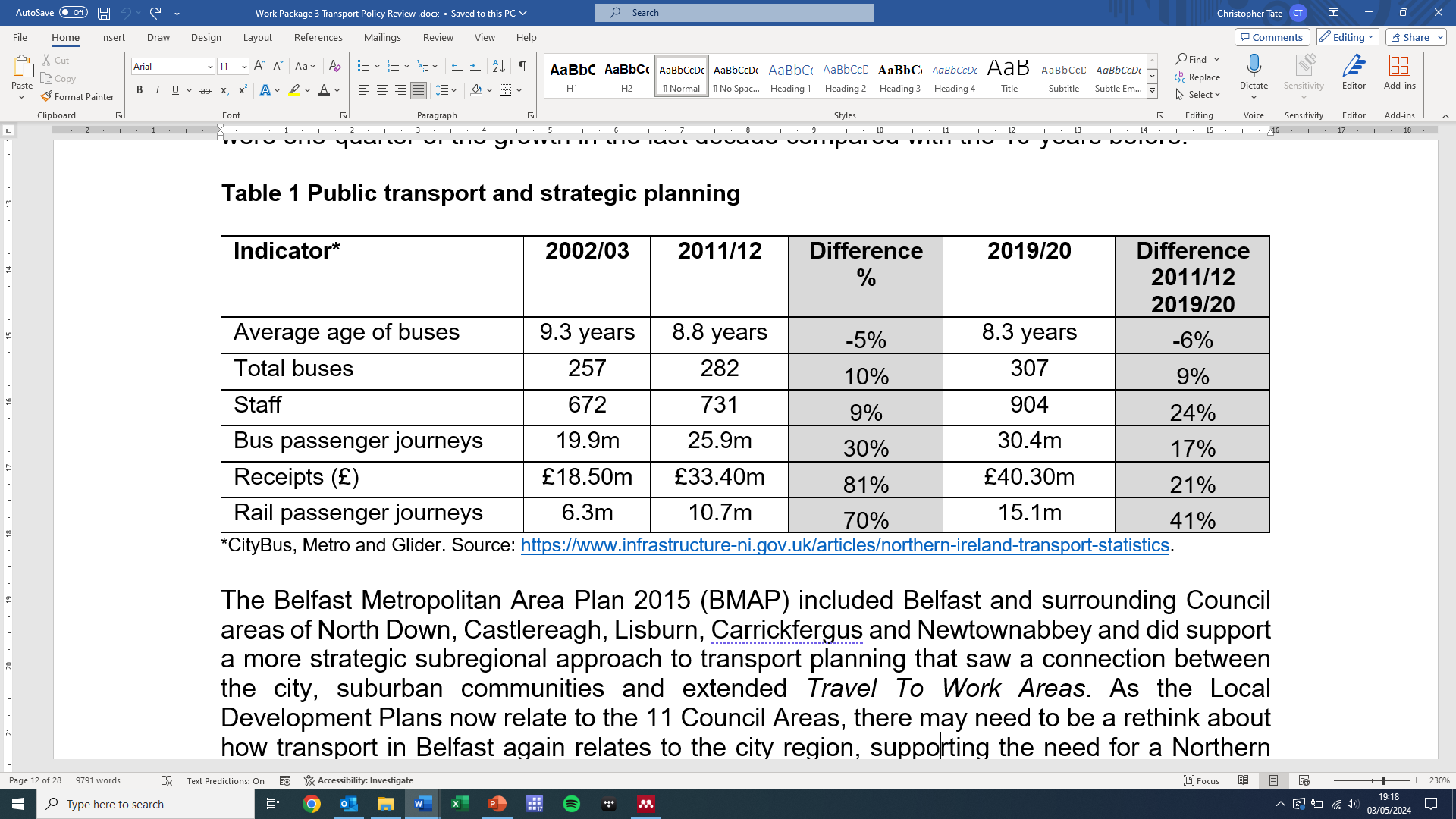


* Citybus, Metro, and Glider

*Source: Source: https://www.infrastructure-ni.gov.uk/articles/northern-ireland-transport-statistics*

An investment of £100m was allocated to support a new rapid transit bus service (Glider), and included 32 articulated buses, employing 109 staff, and a new 24.5km route making use of both dedicated bus lanes and mixed traffic lanes. Weekday services are designed to operate on a 7-9 minute interval, reducing to 4-6 minutes at peak times. Passenger journeys on the Glider grew rapidly from 3.7m journeys in 2018/19 to 7.7m in 2019/20 and passenger receipts also rose from £4.9m to £9.0m over the same period (19).

The Belfast Region City Deal (<https://www.belfastcity.gov.uk/belfastregioncitydeal>) aims to support the Belfast Rapid Transit (BRT) Phase II with new dedicated bus lanes, halts, and upgraded road infrastructure to improve connectivity with the Glider service. A new Glider service has also been planned. This city region policy framework, with costed capital and revenue programmes, will help to facilitate the transition to reduced car use. Phase II of the BRT will also extend to areas outside Belfast city, providing cross-council improvements within the existing transport network.

A key component of rapid transport is the Belfast Transport Hub (<https://www.translink.co.uk/betterconnected/BelfastTransportHub>), which is a significant investment by the Department for Infrastructure at Weaver’s Cross – a multimodal facility with 26 bus stands, 8 railway platforms, cycle and taxi provision – designed to cater for more than 14m passenger journeys per annum, an increase from the current 8m per year. It is also important to note that major investments in road infrastructure have seen a shift in focus in order to strengthen its impact on place making, community health and wellbeing as well as on climate change.

### A.4.6 Investment in active transport

The Department for Infrastructure (DfI) has developed a range of multi-modal initiatives to promote active travel. For example, the Bicycle Strategy for Northern Ireland (20) works through a three-pillar approach based on: i) building a comprehensive network for cycling; ii) supporting cyclists; and iii) promoting cycling as a mode of everyday travel. Under the building infrastructure pillar, the first urban network for Belfast was launched for publication by DfI in 2017 (21). In summary, the draft strategy aims over the next 25 years (from 2017) to get to a point where over 12% of all daily trips in the city would be made by bicycle. The objectives are: to develop a comprehensive bicycle network; bring cycle routes within the reach of most people within the city; ensure a consistent level of service in the design of safe infrastructure; encourage use of the bicycle by increasing the amount of bicycle parking facilities; and create more cycling education programmes.

The DfI also aims to strengthen walking infrastructure with its Strategic Plan for Greenways (22), the overall aim of which is to: improve the connections between greenways; promote better health through increased exercise; improve the safety of Belfast’s greenway network; and improve accessibility to the greenway network especially for disadvantaged communities. The strategy proposes 27 greenways, covering 1036 miles at a cost of £149.5m.

# Part B – Car emissions

## B.1 Greenhouse gas emissions

In the UK, the average petrol car emitted 0.164 equivalent kilograms of CO_2_ (kgCO_2_e) per km, while the average diesel car emitted 0.170kgCO_2_e/km (23). Based on an average journey length of 11.39km, the average emissions per journey for a petrol and diesel car would be 1.87kgCO_2_e and 1.94kgCO_2_e, respectively. Based on an average of 583 journeys made by car per person, each petrol and diesel car emitted on average approximately 1,089kgCO_2_e and 1,129kgCO_2_e of total emissions in 2021, respectively (see Table S1).

Table S1: Average CO_2_ emissions (per car)

|  | % of car market share | Average journeys made per year | Average emissions per km (kgCO_2_e) | Average car journey length (km) | Average emissions per journey (kgCO_2_e) | Total emissions per year (kgCO_2_e) |
| --- | --- | --- | --- | --- | --- | --- |
| Petrol car | 59% | 583 | 0.164 | 11.39 | 1.86796 | 1,089.02 |
| Diesel car | 35% | 583 | 0.170 | 11.39 | 1.9363 | 1,128.86 |

If 59% of the distance travelled in Belfast was by petrol car (~851m kilometres), we estimate that 139,509tCO_2_e were generated in 2021 by petrol cars (Table S2 in the Appendix Part B.1). For diesel cars, which account for 35% of cars (and therefore ~505m kilometres travelled), this is equal to 85,787tCO_2_e. Based on 2020/21 prices, the UK Government values carbon emissions at £241 per ton of CO_2_ (24). Therefore, petrol and diesel car emissions can be valued at £33.6m and £20.7m in 2021, respectively. Reducing the number of car journeys made by one day per week (i.e., 52 across one year) would result in 37.9m fewer kilometres travelled by petrol car and 22.5m kilometres by diesel car. The combined resultant drop in emissions would be 10,048tCO_2_e which is valued at ~£2.42m (see Table S2).

Table S2: Value of CO_2_ emissions

|  | Unit |  |
| --- | --- | --- |
| **CO_2_ emissions (per year)** | tCO_2_e |  |
| *Petrol car* |  | 139,508.92 |
| *Diesel car* |  | 85,787.32 |
| *Total** |  | 225,296.23 |
| **Price of carbon** | £/tCO_2_e | 241 |
| **Value of CO_2_ emissions (per year)** | £ |  |
| *Petrol car* |  | 33,621,649.23 |
| *Diesel car* |  | 20,674,742.97 |
| *Total** |  | 54,296,392.20 |
| **Car journey reductions (per year)** |  |  |
| 50% of population aged ≥ 18 with car access | n | 108,564 |
| 1 day less driving per week (per person) | km | 592.28 |
| 1 day less driving (50% of population with car access) | km | 64,299,989.78 |
| *Petrol car* |  | 37,936,993.97 |
| *Diesel car* |  | 22,504,996.42 |
| *Total** |  | 60,441,990.39 |
| **CO_2_ emissions reductions (per year)** | tCO_2_e |  |
| *Petrol car* |  | 6,221.67 |
| *Diesel car* |  | 3,825.85 |
| *Total** |  | 10,047.52 |
| **Value of CO_2_ emissions reductions (per year)** | £ |  |
| *Petrol car* |  | 1,499,421.75 |
| *Diesel car* |  | 922,029.70 |
| *Total** |  | 2,421,451.45 |
| * Calculated as the sum of petrol and diesel cars, excluding hybrid and electric vehicles | | |

## B.2 Particulate matter emissions

In 2021, in the UK it was estimated that road transport accounted for 17,529 tons of PM_10_ emissions (or 12% of total PM_10_ emissions) and 10,733 tons of PM_2.5_ emissions (or 13% of total PM_2.5_ emissions) (25). Historical air quality emissions data published by the National Atmospheric Emissions Inventory (NAEI) (26) indicated that passenger cars were responsible for a combined 3,300 tons of PM_10_ and PM_2.5_ in 2021. The UK Government’s central damage cost value (in 2022 prices) for PM_10_ and PM_2.5_ is £74,769 per ton (27). The source sector damage cost of road transport is slightly higher at £84,548 per ton. As such, the source sector damage cost values for UK road transport PM_10_ and PM_2.5_ emissions in 2021 were ~£1.48bn and ~£908m, respectively.

### B.2.1 Exhaust emissions

Data published by the NAEI indicated that PM (PM_10_ = PM_2.5_) hot exhaust emissions for petrol and diesel cars on urban roads were 1mg/km and 7mg/km in 2021, respectively (28). This resulted in a combined annual average of PM_10_ and PM_2.5_ emissions of 13,281mg and 92,965mg for each petrol and diesel car in Northern Ireland, respectively (see Table S3)

Table S3: Average exhaust particulate matter emissions (per car)

|  | Average journeys made per year | Emissions per km (mg) | Average car journey length (km) | Average emissions per journey (mg) | Total emissions per year (mg) |
| --- | --- | --- | --- | --- | --- |
| PM_10_ |  |  |  |  |  |
| *Petrol car* | 583 | 1 | 11.39 | 11.39 | 6,640.37 |
| *Diesel car* | 583 | 7 | 11.39 | 79.73 | 46,482.59 |
| PM_2.5_ |  |  |  |  |  |
| *Petrol car* | 583 | 1 | 11.39 | 11.39 | 6,640.37 |
| *Diesel car* | 583 | 7 | 11.39 | 79.73 | 46,482.59 |

In Belfast, we estimate that exhaust emissions from petrol and diesel cars were responsible for 1.70tPMe and 7.06tPMe, respectively. Reducing the number of car journeys made by one day per week for half of the population aged ≥ 18 years with access to a petrol or diesel car will result in a decrease of PM_10_ and PM_2.5_ exhaust emissions across both petrol and diesel cars of 0.4 tPMe which has a source sector damage cost value of £33,054 (see Table S4).

Table S4: Value of exhaust particulate matter emissions

|  | Unit |  |
| --- | --- | --- |
| **PM_10_ emissions (per year)** | tPM_10_e |  |
| *Petrol car* |  | 0.85 |
| *Diesel car* |  | 3.53 |
| *Total* |  | 4.38 |
| **Price of PM_10_** | £/tPM_10_e | 84,548 |
| **Value of PM_10_ emissions (per year)** | £ |  |
| *Petrol car* |  | 71,921.95 |
| *Diesel car* |  | 298,658.95 |
| *Total* |  | 370,580.90 |
| **PM_2.5_ emissions (per year)** | tPM_2.5_e |  |
| *Petrol car* |  | 0.85 |
| *Diesel car* |  | 3.53 |
| *Total* |  | 4.38 |
| **Price of PM_2.5_** | £/tPM_2.5_e | 84,548 |
| **Value of PM_2.5_ emissions (per year)** | £ |  |
| *Petrol car* |  | 71,921.95 |
| *Diesel car* |  | 298,658.95 |
| *Total* |  | 370,580.90 |
| **Car journey reductions (per year)** |  |  |
| 50% of population aged ≥ 18 with car access | n | 108,564 |
| 1 day less driving per week (per person) | km | 592.28 |
| 1 day less driving (50% of population with car access) | km | 64,299,989.78 |
| *Petrol car* |  | 37,936,993.97 |
| *Diesel car* |  | 22,504,996.42 |
| *Total** |  | 60,441,990.39 |
| **PM_10_ emissions reductions (per year)** | tPM_10_e |  |
| *Petrol car* |  | 0.04 |
| *Diesel car* |  | 0.16 |
| *Total* |  | 0.20 |
| **Value of PM_10_ emissions reductions (per year)** | £ |  |
| *Petrol car* |  | 3,207.50 |
| *Diesel car* |  | 13,319.27 |
| *Total* |  | 16,526.76 |
| **PM_2.5_ emissions reductions (per year)** | tPM_2.5_e |  |
| *Petrol car* |  | 0.04 |
| *Diesel car* |  | 0.16 |
| *Total* |  | 0.20 |
| **Value of PM_2.5_ emissions reductions (per year)** | £ |  |
| *Petrol car* |  | 3,207.50 |
| *Diesel car* |  | 13,319.27 |
| *Total* |  | 16,526.76 |
| **Value of PM_10_ and PM_2.5_ exhaust emissions reductions (per year)** | £ | 33,053.53 |
| * Calculated as the sum of petrol and diesel cars, excluding hybrid and electric vehicles | | |

### B.2.2 Non-exhaust emissions

Emission factors for PM10 from tyre wear, brake wear, and road abrasion for different vehicle types in urban, rural and motorway settings are shown in Table S5.

Table S5: Emission factors for PM_10_ from non-exhaust emissions (mg PM_10_/km)

|  |  | Tyre wear | Brake wear | Road abrasion |
| --- | --- | --- | --- | --- |
| Cars | Urban | 8.7 | 11.7 | 7.5 |
|  | Rural | 6.8 | 5.5 |  |
|  | Motorway | 5.8 | 1.4 |  |
| LGVs | Urban | 13.8 | 18.2 | 7.5 |
|  | Rural | 10.7 | 8.6 |  |
|  | Motorway | 9.2 | 2.1 |  |
| Rigid HGVs | Urban | 20.7 | 51.0 | 38.0 |
|  | Rural | 17.4 | 27.1 |  |
|  | Motorway | 14.0 | 8.4 |  |
| Artic HGVs | Urban | 47.1 | 51.0 | 38.0 |
|  | Rural | 38.2 | 27.1 |  |
|  | Motorway | 31.5 | 8.4 |  |
| Buses | Urban | 21.2 | 53.6 | 38.0 |
|  | Rural | 17.4 | 27.1 |  |
|  | Motorway | 14.0 | 8.4 |  |
| Motorcycles | Urban | 3.7 | 5.8 | 3.0 |
|  | Rural | 2.9 | 2.8 |  |
|  | Motorway | 2.5 | 0.7 |  |
| Source: *Air Quality Expert Group. Non-Exhaust Emissions from Road Traffic [Internet]. Department for Environment, Food and Rural Affairs; 2019. Available from:* [*https://uk-air.defra.gov.uk/assets/documents/reports/cat09/1907101151_20190709_Non_Exhaust_Emissions_typeset_Final.pdf*](https://uk-air.defra.gov.uk/assets/documents/reports/cat09/1907101151_20190709_Non_Exhaust_Emissions_typeset_Final.pdf) | | | | |

PM10 to PM2.5 conversion factors for tyre wear, brake wear and road abrasion are shown in Table S6.

Table S6: Fraction of PM10 emitted as PM2.5 for non-exhaust traffic emissions

|  | PM2.5 |
| --- | --- |
| Tyre wear | 0.7 |
| Brake wear | 0.4 |
| Road abrasion | 0.54 |

Combined average PM_10_ and PM_2.5_ non-exhaust emission factors from tyre wear, break wear, and road abrasion for vehicles in the UK at typical urban speeds were estimated to be 27.9mg/km and 14.9mg/km, respectively (29). Based on an average car journey length of 11.39km, each car emitted 318mgPM_10_e and 170mgPM_2.5_e per journey. Per year, this is equivalent to 185,266mgPM_10_e and 98,942mgPM_2.5_e (see Table S7).

Table S7: Average non-exhaust particulate matter emissions (per car)

|  | Average journeys made per year | Emissions per km (mg) | Average car journey length (km) | Average emissions per journey (mg) | Total emissions per year (mg) |
| --- | --- | --- | --- | --- | --- |
| **PM_10_** |  |  |  |  |  |
| *Tyre wear* | 583 | 8.7 | 11.39 | 99.093 | 57,771.219 |
| *Brake wear* | 583 | 11.7 | 11.39 | 133.263 | 77,692.329 |
| *Road abrasion* | 583 | 7.5 | 11.39 | 85.425 | 49,802.775 |
| **Combined** |  | **27.9** |  | **317.78** | **185,266.32** |
| **PM_2.5_*** |  |  |  |  |  |
| *Tyre wear* | 583 | 6.1 | 11.39 | 69.479 | 40,506.26 |
| *Brake wear* | 583 | 4.7 | 11.39 | 53.533 | 31,209.74 |
| *Road abrasion* | 583 | 4.1 | 11.39 | 46.699 | 27,225.52 |
| **Combined** |  | **14.9** |  | **169.71** | **98,941.51** |
| * Based on the following PM_10_ to PM_2.5_ conversion factors: tyre wear – 0.7; brake wear – 0.4; road abrasion – 0.54 (29). | | | | | |

As highlighted above, reducing the number of car journeys made by one day per week would result in a decrease of 60.4m kilometres travelled for half of the population aged ≥ 18 years with access to a petrol or diesel car. We estimate that this would generate a reduction in PM_10_ emissions of 1.7 tons, and PM_2.5_ emissions of 0.9 tons. The combined resultant drop in emissions of PM_10_ and PM_2.5_ are valued at £218,719 (see Table S8).

Table S8: Value of non-exhaust particulate matter emissions

|  | Unit |  |
| --- | --- | --- |
| **PM_10_ emissions (per year)** | tPM_10_e |  |
| *Tyre wear* |  | 11.79 |
| *Break wear* |  | 15.86 |
| *Road abrasion* |  | 10.16 |
| *Total* |  | 37.81 |
| **Price of PM_10_** | £/tPM_10_e | 84,548 |
| **Value of PM_10_ emissions (per year)** | £ |  |
| *Tyre wear* |  | 996,911.38 |
| *Break wear* |  | 1,340,673.93 |
| *Road abrasion* |  | 859,406.37 |
| *Total* |  | 3,196,991.68 |
| **PM_2.5_ emissions (per year)** | tPM_2.5_e |  |
| *Tyre wear* |  | 8.27 |
| *Break wear* |  | 6.37 |
| *Road abrasion* |  | 5.56 |
| *Total* |  | 20.19 |
| **Price of PM_2.5_** | £/tPM_2.5_e | 84,548 |
| **Value of PM_2.5_ emissions (per year)** | £ |  |
| *Tyre wear* |  | 698,983.84 |
| *Break wear* |  | 538,561.32 |
| *Road abrasion* |  | 469,808.81 |
| *Total* |  | 1,707,353.98 |
| **Car journey reductions (per year)** |  |  |
| 50% of population aged ≥ 18 with car access | n | 108,564 |
| 1 day less driving per week (per person) | km | 592.28 |
| 1 day less driving (50% of population with car access) | km | 64,299,989.78 |
| *Petrol car* |  | 37,936,993.97 |
| *Diesel car* |  | 22,504,996.42 |
| *Total** |  | 60,441,990.39 |
| **PM_10_ emissions reductions (per year)** | tPM_10_e |  |
| *Tyre wear* |  | 0.523 |
| *Break wear* |  | 0.707 |
| *Road abrasion* |  | 0.453 |
| *Total* |  | 1.686 |
| **Value of PM_10_ emissions reductions (per year)** | £ |  |
| *Tyre wear* |  | 44,459.17 |
| *Break wear* |  | 59,789.92 |
| *Road abrasion* |  | 38,326.87 |
| *Total* |  | 142,575.96 |
| **PM_2.5_ emissions reductions (per year)** | tPM_2.5_e |  |
| *Tyre wear* |  | 0.369 |
| *Break wear* |  | 0.284 |
| *Road abrasion* |  | 0.248 |
| *Total* |  | 0.901 |
| **Value of PM_2.5_ emissions reductions (per year)** | £ |  |
| *Tyre wear* |  | 31,172.52 |
| *Break wear* |  | 24,018.17 |
| *Road abrasion* |  | 20,952.02 |
| *Total* |  | 76,142.72 |
| **Value of PM_10_ and PM_2.5_ non-exhaust emissions reductions (per year)** | £ | 218,718.67 |
| * Calculated as the sum of petrol and diesel cars, excluding hybrid and electric vehicles | | |

## B.3 Nitrogen oxide emissions

The UK Government values the road transport source sector damage cost of NO_X_ at £11,682 per ton (in 2022 prices) (27). It was estimated that in 2021, road transport was responsible for 27% (or 184,893 tons) of total NO_X_ emissions in the UK (30). This is equivalent to ~£2.16bn in source sector damage costs.

NAEI data indicate that NO_X_ hot exhaust emissions for petrol and diesel cars in urban areas were 0.065g/km and 0.544g/km in 2021, respectively (28). Based on this, we estimate that in Northern Ireland, the average petrol and diesel car emitted 0.740 gNO_X_e and 6.196 gNO_X_e per journey in 2021, respectively. Per year, this totals 432gNO_X_e for petrol cars and 3,612gNO_X_e for diesel cars (see Table S9).

Table S9: Average NO_X_ emissions (per car)

|  | % of car market share | Average journeys made per year | Average emissions per km (gNO_X_e) | Average car journey length (km) | Average emissions per journey (gNO_X_e) | Total emissions per year (gNO_X_e) |
| --- | --- | --- | --- | --- | --- | --- |
| Petrol car | 59% | 583 | 0.065 | 11.39 | 0.74035 | 431.62 |
| Diesel car | 35% | 583 | 0.544 | 11.39 | 6.19616 | 3,612.36 |

We estimate that 55tNO_X_e was generated by petrol cars and 275tNO_X_e by diesel cars in 2021. Reducing car usage by one day per week for half the population aged ≥ 18 years with access to a petrol or diesel car will result in a reduction of 14.71 tNO_X_e which is valued at £171,826 (see Table S10).

Table S10: Value of exhaust NO_x_ emissions

|  | Unit |  |
| --- | --- | --- |
| **NO_X_ emissions (per year)** | tNO_X_e |  |
| *Petrol car* |  | 55.29 |
| *Diesel car* |  | 274.52 |
| *Total** |  | 329.81 |
| **Price of nitrogen oxide** | £/tNO_X_e | 11,682 |
| **Value of NO_x_ emissions (per year)** | £ |  |
| *Petrol car* |  | 645,934.80 |
| *Diesel car* |  | 3,206,935.73 |
| *Total** |  | 3,852,870.53 |
| **Car journey reductions (per year)** |  |  |
| 50% of population aged ≥ 18 with car access | n | 108,564 |
| 1 day less driving per week (per person) | km | 592.28 |
| 1 day less driving (50% of population with car access) | km | 64,299,989.78 |
| *Petrol car* |  | 37,936,993.97 |
| *Diesel car* |  | 22,504,996.42 |
| *Total** |  | 60,441,990.39 |
| **NO_X_ emissions reductions (per year)** | tNO_X_e |  |
| *Petrol car* |  | 2.47 |
| *Diesel car* |  | 12.24 |
| *Total** |  | 14.71 |
| **Value of NO_X_ emissions reductions (per year)** | £ |  |
| *Petrol car* |  | 28,806.70 |
| *Diesel car* |  | 143,019.43 |
| *Total** |  | 171,826.13 |
| * Calculated as the sum of petrol and diesel cars, excluding hybrid and electric vehicles | | |

# References

1. Northern Ireland Statistics and Research Agency. 2022 Mid-Year Population Estimates for Northern Ireland [Internet]. 2023. Available from: https://www.nisra.gov.uk/publications/2022-mid-year-population-estimates-northern-ireland

2. Northern Ireland Statistics and Research Agency. Employee earnings in NI 2023 [Internet]. 2023. Available from: https://www.nisra.gov.uk/publications/employee-earnings-ni-2023

3. Northern Ireland Statistics and Research Agency. 2021 Census [Internet]. 2021. Available from: https://www.nisra.gov.uk/statistics/census/2021-census

4. Hensher DA. Hypothetical bias, choice experiments and willingness to pay. Transp Res Part B Methodol. 2010;44(6):735–52.

5. Hausman J. Contingent Valuation: From Dubious to Hopeless. J Econ Perspect. 2012;26(4):43–56.

6. Harrison GW. Experimental Evidence on Alternative Environmental Valuation Methods. Environ Resour Econ. 2006;34(1):125–62.

7. Cummings RG, Taylor LO. Unbiased Value Estimates for Environmental Goods: A Cheap Talk Design for the Contingent Valuation Method. Am Econ Rev. 1999;89(3):649–65.

8. List JA. Do Explicit Warnings Eliminate the Hypothetical Bias in Elicitation Procedures? Evidence from Field Auctions for Sportscards. Am Econ Rev [Internet]. 2001 Dec;91(5):1498–507. Available from: http://www.aeaweb.org/aer/

9. Murtagh B, Garcia L, Hunter RF. Developing system-level interventions to reduce car dependency for improved population health in Belfast: Policy mapping and socio-technical transitions. Belfast: Queen’s University Belfast; 2023.

10. Department for Regional Development (DRD). Regional Development Strategy for Northern Ireland 2035. Belfast: DRD; 2010.

11. Department for Infrastructure (DfI). Strategic Planning Policy Statement Northern Ireland. Beflast: DfI; 2015.

12. Department of the Environment (NI). Planning Policy Statement 3: Access, Movement and Parking. Belfast: The Planning Service; 2005.

13. Department of the Environment (NI). Planning Policy Statement 13: Transportation and Land Use. Belfast: The Planning Service; 2005.

14. Department for Infrastructure (DfI). Development Control Advice Note 15: Vehicular Access Standards. Belfast: The Planning Service; 2019.

15. Department for Regional Development (DRD). Ensuring a Sustainable Transport Future: A New Approach to Regional Transportation. Belfast: DRD; 2011.

16. Department for Regional Development (DRD). Belfast on the Move: Transport Masterplan for Belfast City Centre Post Implementation Impact Study. Belfast: DRD; 2014.

17. Department for Regional Development (DRD). Regional Transportation Strategy for Northern Ireland 2002 – 2012. Belfast: DRD; 2002.

18. Department for Regional Development (DRD). Regional Development Strategy for Northern Ireland 2015. Belfast: DRD; 2001.

19. Department for Infrastructure (DfI). Northern Ireland Transport Statistics 2019-20. Belfast: DfI; 2020.

20. Department for Regional Development (DRD). Northern Ireland Changing Gear: A Bicycle Strategy for Northern Ireland. Belfast: DRD; 2015.

21. Department for Infrastructure (DfI). Draft Belfast Bicycle Network 2017 Cycling Unit Consultation Document. Belfast: DfI; 2017.

22. Department for Infrastructure. Exercise, Explore, Enjoy: A Strategic Plan for Greenways [Internet]. Belfast; 2016. Available from: https://www.infrastructure-ni.gov.uk/publications/exercise-explore-enjoy-strategic-plan-greenways

23. Department for Energy Security and Net Zero. Greenhouse gas reporting: conversion factors 2023 [Internet]. UK Government; 2023. Available from: https://www.gov.uk/government/publications/greenhouse-gas-reporting-conversion-factors-2023

24. HM Treasury. The Green Book: Appraisal and Evaluation in Central Government [Internet]. UK Government; 2023. Available from: https://www.gov.uk/government/publications/the-green-book-appraisal-and-evaluation-in-central-government/the-green-book-2020#fn:32

25. Department for Environment Food & Rural Affairs. Emissions of air pollutants in the UK – Particulate matter (PM10 and PM2.5) [Internet]. UK Government; 2023. Available from: https://www.gov.uk/government/statistics/emissions-of-air-pollutants/emissions-of-air-pollutants-in-the-uk-particulate-matter-pm10-and-pm25

26. National Atmospheric Emssions Inventory. Historical Air Quality Emissions Data [Internet]. 2023. Available from: https://naei.beis.gov.uk/data/

27. Department for Environment Food & Rural Affairs. Air quality appraisal: damage cost guidance [Internet]. UK Government; 2023. Available from: https://www.gov.uk/government/publications/assess-the-impact-of-air-quality/air-quality-appraisal-damage-cost-guidance#annex-a

28. National Atmospheric Emssions Inventory. Fleet weighted road transport emission factors 2021 [Internet]. 2022. Available from: https://naei.beis.gov.uk/data/ef-transport

29. Air Quality Expert Group. Non-Exhaust Emissions from Road Traffic [Internet]. Department for Environment, Food and Rural Affairs; 2019. Available from: https://uk-air.defra.gov.uk/assets/documents/reports/cat09/1907101151_20190709_Non_Exhaust_Emissions_typeset_Final.pdf

30. Department for Environment Food & Rural Affairs. Emissions of air pollutants in the UK – Nitrogen oxides (NOx) [Internet]. UK Government; 2023. Available from: https://www.gov.uk/government/statistics/emissions-of-air-pollutants/emissions-of-air-pollutants-in-the-uk-nitrogen-oxides-nox#:~:text=Emissions of nitrogen oxides have,cent between 2020 and 2021.
